# Molecular Interactions of the Min Protein System Reproduce Spatiotemporal Patterning in Growing and Dividing *Escherichia coli* Cells

**DOI:** 10.1371/journal.pone.0128148

**Published:** 2015-05-27

**Authors:** James C. Walsh, Christopher N. Angstmann, Iain G. Duggin, Paul M. G. Curmi

**Affiliations:** 1 School of Physics, University of New South Wales, Sydney NSW 2052, Australia; 2 The ithree institute, University of Technology, Sydney NSW 2007, Australia; 3 School of Mathematics and Statistics, University of New South Wales, Sydney NSW 2052, Australia; Centre National de la Recherche Scientifique, Aix-Marseille Université, FRANCE

## Abstract

Oscillations of the Min protein system are involved in the correct midcell placement of the divisome during *Escherichia coli* cell division. Based on molecular interactions of the Min system, we formulated a mathematical model that reproduces Min patterning during cell growth and division. Specifically, the increase in the residence time of MinD attached to the membrane as its own concentration increases, is accounted for by dimerisation of membrane-bound MinD and its interaction with MinE. Simulation of this system generates unparalleled correlation between the waveshape of experimental and theoretical MinD distributions, suggesting that the dominant interactions of the physical system have been successfully incorporated into the model. For cells where MinD is fully-labelled with GFP, the model reproduces the stationary localization of MinD-GFP for short cells, followed by oscillations from pole to pole in larger cells, and the transition to the symmetric distribution during cell filamentation. Cells containing a secondary, GFP-labelled MinD display a contrasting pattern. The model is able to account for these differences, including temporary midcell localization just prior to division, by increasing the rate constant controlling MinD ATPase and heterotetramer dissociation. For both experimental conditions, the model can explain how cell division results in an equal distribution of MinD and MinE in the two daughter cells, and accounts for the temperature dependence of the period of Min oscillations. Thus, we show that while other interactions may be present, they are not needed to reproduce the main characteristics of the Min system *in vivo*.

## Introduction

Bacterial cell division requires the precise placement and timing of the FtsZ division ring to produce two viable daughter cells [1]. To date, two separate negative regulators of the location of the FtsZ division ring in *Escherichia coli* have been identified: nucleoid occlusion and the Min protein system [1]. The Min system prevents ring formation at the poles of the rod-shaped cells by locally inhibiting FtsZ polymerization [2, 3]. To achieve this, the distribution of Min proteins forms an oscillatory spatiotemporal pattern with proteins localizing at one pole of the cell, then the other, leaving a bare zone at the centre of the cell where the divisome will form. Inhibition of the Min protein system results in asymmetric division [1] and leads to a proportion of contractile rings forming at the end caps, with subsequent formation of mini-cells [4].

The spatiotemporal patterns formed by Min proteins in bacterial cells have been observed in detail using fluorescence microscopy [5]. Three studies have investigated how Min patterning changes as a function of cell length in *E*. *coli* over a normal division cycle by tracking the distribution of GFP labelled MinD [6–8]. Although the results were qualitatively similar, significant differences were observed that appear to arise from differences in experimental conditions (the extent of MinD labelling: fully-labelled [6] versus partially-labelled [7] and the overexpression of Min proteins in the fully-labelled case [6, 9]). Differences included the period of Min oscillation and the critical cell length for cell division. The difference in oscillation period due to labelling has also been reported independently [10]. The differences between spatiotemporal patterns observed in the various reports [6–8] are likely to come from variations in Min protein expression levels [9] and the effects of labelling of MinD on one or more of MinD’s interactions and hence its function. The latter is supported by the disappearance of the transition from stationary to oscillating patterning (seen only when MinD is fully-labelled) when MinE is fluorescently labelled instead of MinD [6].

Throughout and following cell division Min protein patterning is continuously maintained [6, 7]. To achieve this, approximately equal quantities of two Min proteins, MinD and MinE, must remain on either side of the septum following binary fission. While cell division is not sensitive to the absolute concentration of Min proteins (with cells overexpressing the Min operon by six- to sevenfold dividing as per wild type), it is sensitive to the ratio between MinE and MinD with a two-fold reduction in MinE relative to MinD prohibiting cell division [11].

*In vitro* experiments have shown that a minimal MinE-MinD ATPase system is capable of spontaneously producing spatiotemporal patterns. Combining purified MinE and MinD over an artificial planar lipid bilayer in the presence of ATP, these Min proteins spontaneously produce travelling wave patterns [12]. The small number of components required for patterning emphasizes that the Min system is amenable to mathematical modelling that should provide insight into cellular patterning and cell division without the need to introduce complex regulatory mechanisms. This has been reinforced *in vivo* with the deletion of MinC, the only known interaction partner of MinD and MinE, having little effect on oscillations [5].

The basic molecular interactions of the Min system are well understood. MinD is an ATPase that is able to bind to the lipid bilayer when it is in the MinD.ATP state [13]. MinD.ATP is able to dimerise [14] once bound to the membrane [15, 16]. Dimerisation may stabilize the membrane-bound state, giving rise to cooperativity under steady state conditions [13]. MinE is a bistable dimeric protein [17] that binds to both MinD [18] and transiently to the membrane when in its active state [19]. When bound to MinD, MinE stimulates the MinD ATPase, producing MinD.ADP which is then released from the membrane [20]. Thus, MinD cycles between the cytoplasm and the membrane in a cooperative, ATP dependent manner. MinE follows behind MinD on the membrane, effectively stripping MinD from the surface [21].

Several mathematical models for the Min system in *E*. *coli* have been proposed [22]. Each introduces non-linear terms to create spatio-temporal patterning. How the wave shape of the Min system changes throughout the cell cycle is likely to be heavily dependent on these non-linear reactions [23]. Current models of the Min system fall roughly into two classes depending on the effective non-linear interactions they introduce to induce patterning: cooperative attachment and aggregation current models [22].

Cooperative attachment systems introduce non-linearities into the binding and release reactions. The seminal example of this was proposed by Huang [24]. This model has an increase in the rate of MinD binding to the membrane proportional to the amount of MinD already bound to the membrane in both MinD monomer and MinD/E complex form. While the binding of MinD to phospholipid vesicles is cooperative [13], non-hydrolysable analogues of ATP show that this is a two step process [25]. Many variations of this model have been published, including transformation to a stochastic model [26] and introducing finite binding locations for MinD [27]. This finite binding model also has a stochastic analogue where binding is mediated by neighbouring binding sites [28]. Other variations include increasing the binding rate only as a function of bound MinD monomers, which has been simulated both stochastically [29] and deterministically [30], as well as incorporating MinE membrane binding [31]. Combined, these models have been able to recreate the majority of critical behaviour of the Min system, albeit each using independent parameter sets. Described phenomena include *in vitro* patterning [31], discretely observing the changes in patterning during cell division [8], and by modifying protein concentrations, the critical transitions of the fully-labelled Min system [31].

Models utilising aggregation currents primarily rely on anomalous diffusion of proteins bound to the membrane to cause the non-linearities required to give rise to spatial patterns. A classic example was developed by Meacci [32]. This model is built on two assumptions: there are a limited number of binding locations for MinD on the membrane and that once bound, MinD is attracted to other membrane-bound MinD molecules. This anisotropic diffusion is taken to be of the Cahn Hilliard form [33]. In this model, the range of MinD self-interactions is approximately 350 *nm*. Ionic shielding ensures that there is no electrostatic interaction that could act over such distances; being orders of magnitude higher than reasonable values [34]. While membrane mediated forces can span such distances [35] and MinD has been shown to deform vesicles [25], there is currently no evidence that the Min protein system uses this method of interaction. This model has been applied to multiple phenotypes including nearly spherical cells [36]. It has also been converted to a stochastic form [6].

In this paper, we propose a model based on potential molecular interactions of the Min proteins which are consistent with current experimental observations. In this model, membrane-binding and dimerisation of MinD provides the two-step non-linear reaction required to give rise to the observed time dependent patterning of the Min system in *E*. *coli*. The distribution of MinD as a function of cell length calculated from the model qualitatively matches experimentally observed distributions. The model accounts for changes in patterning just prior to cell division and for the equipartitioning of MinD and MinE following cell division, which maintains the same MinE to MinD ratio as the parent cell. Discrepancies between experimental observations using fully-labelled and partially-labelled MinD can be accounted for by reducing the rate constant controlling MinD ATPase and MinDE heterotetramer dissociation for the fully-labelled protein by a factor of four. Similarly, the model accurately accounts for the variation in the Min oscillation period as a function of temperature by multiplying the rate constant controlling the ATPase reaction by a Boltzmann factor. Specific features of the Min system for fully-labelled MinD, such as stationary MinD distribution in newly divided cells (length < 2.75 *μm*), and for partially-labelled MinD, such as midcell antinodes, arise naturally from the model. In so doing, we show that while polymerization and heterogeneous reactions may be present, they are unnecessary to produce the main characteristics of the Min system *in vivo*.

## Materials and Methods

### Model Formulation

Combining the mechanics for MinD dimerisation and MinE interactions, we have developed a partial differential equation (PDE) model. In the equations, the concentrations of the proteins MinD and MinE are represented by single letters: *D* and *d*, and *E* and *e*, respectively. Upper case letters (*D*, *E*) denote species in solution while lower case letters (*d*, *e*) are bound to the membrane. Dimers are indicated by a subscript ‘2’.

The binding of MinD to the membrane is cooperative, suggesting MinD self association [13]. Crystal structures of MinD demonstrate it is capable of forming a dimer in solution when ATP is bound, albeit under non-physiological conditions [14]. While MinD may partially dimerise in solution, FRET [15] and yeast two-hybrid [16] experiments show that MinD dimerisation is predominantly a two-step process. First, MinD monomers with ATP bound are able to bind to the membrane. Once bound, they are then able to dimerise which re-enforces the strength of the membrane binding, increasing its stability. This is depicted in the model with MinD monomers in solution (*D*) being able to bind to the membrane (*d*) before they can react to form a dimer (*d*_*2*_)

The interactions of MinE have been characterised to a lesser extent. MinE is always found as a dimer. Crystal [18, 37] and NMR [17, 38] structures show a dramatic change in structure of MinE between its inactive solution state and its active state that is capable of binding MinD to form a MinD/E heterotetramer on the membrane.

There is evidence for the membrane binding of MinE being both a one and two step transition with the MinE dimer in solution (*E*_*2*_) being able to bind directly to both the membrane (*e*_*2*_), as well as membrane-bound MinD dimers to form a heterotetramer [19]. Either of these pathways on its own is sufficient to reproduce the experimental data (see S1 Text for a direct comparison). Thus, there is no gain in having both pathways. We did not include the two pathways in our model as it was not worth the increase in parameter space, and the probable over-fitting that would result. We have simplified our model by assuming that MinE binding to the membrane is the dominant pathway, and hence all MinE first binds to the membrane before reacting with MinD. The importance of MinE membrane binding has also been demonstrated theoretically with MinE processivity being a critical component in other modern models of the Min system [31, 39].

The configuration of the MinE dimer when it is bound to the membrane is not known. It could be active or inactive with respect to its ability to activate MinD and it is possible that the two MinE monomers are conformationally distinct. We assume that when membrane-bound, MinE is in the active conformation but only one of the two MinE monomers is able to interact with membrane-bound MinD at one time. We ignore the possibility of MinE and MinD to form a heteropolymer where both MinE monomers bind to and activate MinD simultaneously. Such polymers are seen in the MinD/E heterotetramer crystal structure [19], however, we note that there is a 90° rotation between successive MinD dimers in the crystal due the four-fold screw axis. This may preclude the formation of such heteropolymers when both MinD and MinE are tethered to the cell membrane.

Whether MinE is able to interact with MinD monomers is also unknown. As the ATP binding domain is part of the dimer interface, it is unlikely that any such interaction would stimulate ATP hydrolysis, however it may still instigate the release of MinD from the membrane. We assume that this is the case. It is possible to exclude this reaction and still have patterning, however, to date, all models without this interaction suffer the same problem as previous models, that is, they fail to reproduce patterning that is consistent with experimental kymographs.

MinE binding to a single site of MinD is sufficient to catalyze the hydrolysis of both ATP molecules in the dimer [40]. MinD hydrolysis causes the complex to dissociate. MinD in its ADP state returns to the cytosol as monomers (*D*) while MinE remains on the membrane as a homodimer (*e*_*2*_). This entire ATP hydrolysis and MinD dissociation process is modelled by a single reaction characterised by the rate constant *ω*_*hydr*_. The ability of two MinE dimers to bind simultaneously to a single MinD dimer is ignored as it is not required for ATPase activation.

We have made further simplifying assumptions to reduce the size of the parameter space of the model. In solution MinD exists in both an active MinD.ATP and an inactive MinD.ADP state. The inclusion of both of these states does not affect simulations greatly, and so, in our model we assumed that all MinD in solution is in the active state. The release of MinD monomers from the membrane was assumed to be essentially instantaneous upon interaction with MinE.

**Fig 1:**
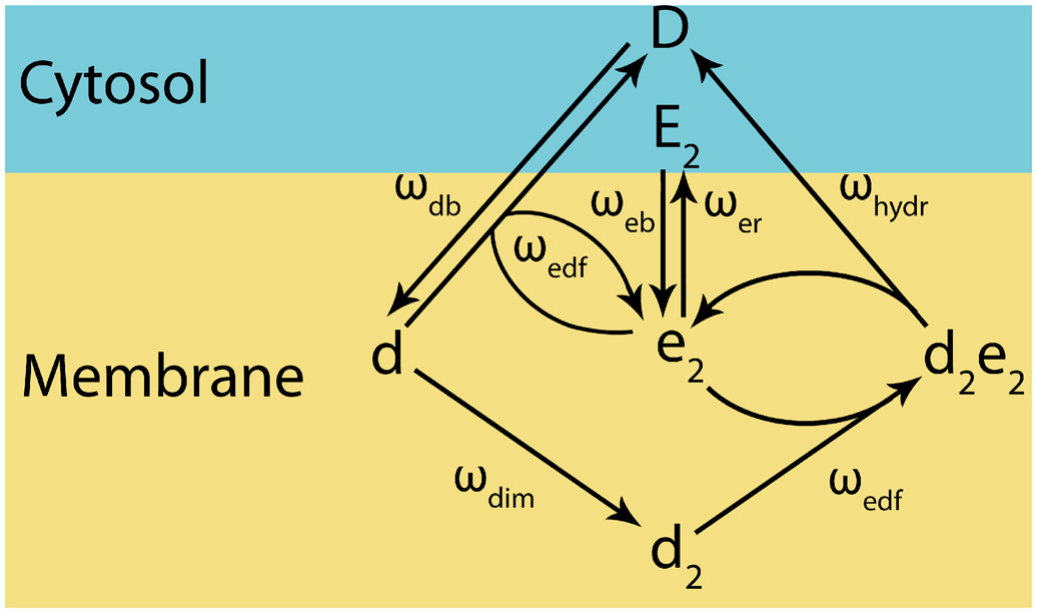
Overview of the Simplified Reactions Underlying the Model. A schematic showing the different states of the system and the reaction pathways between them. *ω* denote the respective rate parameter for each reaction. The value used for each of these parameters and a description of each reaction is shown in Table 1.

To summarise, the model is governed by the following reactions: MinE dimer in solution (*E*_*2*_) is able to bind to the membrane (*e*_*2*_) and, in turn, can be released back into the cytosol. MinD monomer in solution (*D*) can bind to the membrane (*d*). Membrane-bound MinD (*d*) can either form a dimer (*d*_2_) or bind to membrane-bound MinE (*e*_*2*_), which stimulates the MinD monomer’s release from the membrane. The MinD dimer (*d*_*2*_) can form a heterotetramer complex (*d*_*2*_ *e*_*2*_) by binding the membrane-bound MinE homodimer (*e*_*2*_). In the heterotetramer (*d*_*2*_ *e*_*2*_) MinD hydrolyses ATP causing the complex to dissociate with MinD being released into the cytosol as monomers (*D*) while MinE returns to being a membrane-bound homodimer (*e*_*2*_). These simplified reactions are shown schematically in Fig 1.

Where possible, parameters have been constrained to fit experimental measurements. Free parameters were found using a genetic algorithm that rated the fitness of parameter sets by the existence of critical transitions within the relevant cell length ranges. The transitions were: that from the stationary to oscillating patterning at 2.7 *μ*m; the transition from the first to the second order mode at 5.5 *μ*m; and the transition to midcell antinodes in the partially-labelled system at 3 *μ*m. Determining the free parameters was achieved by solving for a reduced one-dimensional version of the equations at fixed lengths 0.25 *μm* either side of each transition. All parameters are unaffected, however, the cytosolic states are approximated as homogeneous radially and a coordinate transform is used to project the membrane diffusion onto a one-dimensional line. The resulting parameters and the reactions they control are summarised in Table 1.

**Table 1:** Rate Parameters and Reactions Summary.

Rate	Reaction	Value	Min^a^	Max^a^	Measured	Units
*ω*_*db*_	MinD binding to membrane	4	0.85	19		*μm s* ^-1^
*ω*_*edf*_	MinD-MinE complex formation	22	8	38		*μm* ^2^ *s* ^-1^
*ω*_dim_	MinD dimerisation	0.002	0.001	0.008		*μm* ^2^ *s* ^-1^
*ω*_*hydr*_	Fully-labelled MinD ATPase & tetramer dissociation	0.12	0.075	0.36		*s* ^-1^
*ω*_*hydr*_	Partially-labelled MinD ATPase & tetramer dissociation	0.5				*s* ^-1^
*ω*_*eb*_	MinE binding to membrane	0.07	0.02	0.35	⪡*ω*_*er*_ · *μm* [19]	*μm s* ^-1^
*ω*_*er*_	MinE release from the membrane	30	15	100	⪢*ω*_*eb*_ · *μm* ^-1^ [19]	*s* ^-1^
*D*_*D*_	Diffusion of labelled MinD in solution	16	4	>1000	~16 [41]	*μm* ^2^ *s* ^-1^
*D*_*D*_	Diffusion of unlabelled MinD in solution	24	4	>1000	~24 [41]^b^	*μm* ^2^ *s* ^-1^
*D*_*E*_	Diffusion of MinE in solution	20	0.625	>1000	~20 [41]^b^	*μm* ^2^ *s* ^-1^
*D*_*m*_	Diffusion of membrane-bound states	0.1	0.02	0.195	~0.2 [41]	*μm* ^2^ *s* ^-1^

^a^Minimal and Maximal values that support first order Min oscillations in the fully labelled system at 3.5 *μm*.

^b^Calculated from experimental measurements on GFP-labelled fusion proteins.

MinE strongly favours its inactive state in solution, with membrane-bound MinE homodimers being highly unstable and hence only existing transiently [17]. This is reflected in our model by having the rate of release of bound MinE homodimers (*ω*_*er*_) exceeding their rate of binding (*ω*_*eb*_). As seen in Table 1, the ratio of these rates is 430 *μm* ^-1^. So at equilibrium in the absence of MinD, 99.75% of MinE will remain in the cytoplasm.

To date, no experiment has been performed to differentiate between the rate at which MinE interacts with membrane-bound MinD monomers versus dimers. With no other information, we assume the same association rate, *ω*_*edf*_, for both species. This approximation also helps to minimize the size of the parameter space of the model.

All reactions in this model will have associated back rates, which have been set to zero so as to simplify the model and reduce the parameter space. There are also other reactions that are may occur (including MinD dimerisation in solution) which are also not included as experimental evidence suggests that they are not the prevalent interactions of the physical system [15, 16].

The diffusion coefficients of the fluorescent cytoplasmic MinD-GFP and MinE-GFP fusion proteins have been measured *in vivo* as 16 and 10 *μm* ^2^ *s* ^-1^, respectively [41]. This value for the MinD-GFP diffusion constant, *D*_*D*_, was used in our simulations of the Min system where all of MinD is labelled.

For cytoplasmic MinE and for unlabelled MinD, the difference in the diffusion coefficient between the GFP labelled and unlabelled protein can be approximated. MinE-GFP is approximately four times the size of the wild type MinE (88 versus 326 amino acids). If both of these proteins were spherical this difference in size would result in wild type MinE having twice the diffusion coefficient measured for MinE-GFP [42], that is, *D*_*E*_ approximately 20 *μm* ^2^ *s* ^-1^. By a similar argument, the unlabelled diffusion constant for MinD, D_*D*_, is approximately 24 *μm* ^2^ *s* ^-1^.

Membrane-bound diffusion coefficients are modelled by a single parameter *D*_*m*_. In reality each membrane-bound species would have a different diffusion coefficient depending on the type of complex within which it resides. In our model, we assume that the diffusion coefficient of each membrane-bound species varies according to the inverse of the number of membrane targeting sequences present in the complex (i.e. MinD monomer, dimer, MinE dimer and the MinDE heterotetramer will have diffusion coefficients of 1, ½, ½, and ¼ D_*m*_, respectively). This relationship was determined experimentally using single molecule measurements of diffusion coefficients for engineered proteins containing one to three pleckstrin homology domains coupled by flexible linkers [43]. The reduced diffusion rate of larger complexes (dimers and tetramers) effectively causes a slight agglomeration of the Min system. Although this is not required for this model to function, it has been used as the basis for other aggregation current models which utilize anomalous diffusion to give rise to Min patterning [22].

As protein synthesis and degradation are not important for patterning during *E*. *coli* cell division [5], the average concentrations of both proteins in the cell were set to constant values, for MinD 1389 *μm* ^-3^ (2.3 *μM)* and for MinE homodimers 486 *μm* ^-3^ (0.8 *μM)* (that is, 972 *μm* ^-3^ of MinE monomers), consistent with reported values [44]. In growing domain simulations we maintained a constant average Min concentration in the cell through spatially homogenous protein production added to cytoplasmic *D* and *E*_*2*_ states for MinD and MinE, respectively. These approximations are commensurate with previous models [24].

We note that the absolute concentrations of MinD and MinE used in our simulations may not correspond to the actual concentrations in the fully-labelled MinD experiments [6], where both MinD and MinE were overexpressed in *E*. *coli* strain JS964 containing the plasmid pAM238 encoding for MinE and GFP-MinD under the control of the lac promoter [45]. Due to overexpression, the actual concentrations may be higher [9], however, they were not measured. Higher concentrations of MinD and MinE can be compensated in our partial differential equation model by rescaling only two parameters: *ω*_*edf*_ and *ω*_dim_ which control the only non-linear terms. Dividing each of these rate constants by the ratio of the actual Min protein concentrations and our assumed values will leave the form of the solutions (as presented in the kymographs) unchanged.

We assume an idealized geometry for an *E*. *coli* cell to be a cylinder with spherical end caps. The radius of the cylinder and end caps was fixed at 0.5 *μm* with the length of the cylinder varying depending on the simulation.

The membrane was taken as the two dimensional manifold given by the boundary of the cytoplasm. This manifold is denoted by **M** in the equations where **x** denotes the position within the cytoplasm. As a result, the Dirac delta function in the equations, *δ*(||**x**—**M**||) defines a thin region of cytoplasm close to the membrane within which aqueous species can interact with the membrane.

The resulting model can be written as a set of partial differential reaction-diffusion equations:

$$\begin{array}{l} \partial_{t}D=\delta \left(\left\|x-M\right\|\right)\left(\omega_{edf}e_{2}\cdot d+2\omega_{hydr}d_{e}e_{2}-\omega_{db}D\right)+D_{D}\nabla^{2}D\\ \partial_{t}E_{2}=\delta \left(\left\|x-M\right\|\right)\left(\omega_{er}e_{2}-\omega_{eb}E_{2}\right)+D_{E}\nabla^{2}E_{2}\\ \partial_{t}d=\omega_{db}D-2\omega_{\text{dim}}d^{2}-\omega_{edf}e_{2}\cdot d+D_{m}\nabla^{2}d\\ \partial_{t}d_{2}=\omega_{\text{dim}}d^{2}-\omega_{edf}e_{2}\cdot d_{2}+\frac{1}{2}D_{m}\nabla^{2}d_{2}\\ \partial_{t}e_{2}=-\omega_{er}e_{2}+\omega_{eb}E_{2}-\omega_{edf}e_{2}\cdot d_{2}+\omega_{hydr}d_{2}e_{2}+\frac{1}{2}D_{m}\nabla^{2}e_{2}\\ \partial_{t}d_{2}e_{2}=\omega_{edf}e_{2}\cdot d_{2}-\omega_{hydr}d_{2}e_{2}+\frac{1}{4}D_{m}\nabla^{2}d_{2}e_{2} \end{array}$$

where we note that *d*_*2*_ *e*_*2*_ represents the concentration of the MinDE heterotetramer (and not the product of the concentrations of MinD and MinD dimers, which is represented by *e*_2_⋅*d*_2_). The robustness of the parameter set (summarised in Table 1) to variation was investigated. Whilst holding the remainder of the parameters constant, each parameter was varied until the system was no longer maintained a definite node at the middle of 3.5 *μm* cell. Minimum and maximum values attained for each parameter are shown in Table 1.

### Solving the PDEs

Solving the partial differential equations utilized the numerical software FlexPDE (version 6.32, PDE Solutions Inc.). Within this software, the cell geometry was reduced to two dimensions by assuming that solutions were cylindrically symmetric about the major axis of the cell. The Dirac delta function on the membrane was approximated by a rectangular function with the step occurring 0.05 *μm* from the boundary. FlexPDE cannot handle the coupling between a three-dimensional volume and a two-dimensional manifold, so the membrane was approximated by a thin three-dimensional shell. A similar approximation has been used in other models [29]. The model parameters were scaled to make the resulting reactions independent of the width of this shell. To do this, for each reaction occurring on the membrane, the concentration of each membrane-bound protein was multiplied by the width of the shell. Each complete reaction was then divided by the width of the shell. Finally each resulting constant was then incorporated into the parameter associated with that reaction for the simulation.

### Growing Cell Simulations

In growing domain experiments, the cells are grown linearly in time by stretching the cylindrical section of the cell. If the equations were solved under these conditions, particles would effectively be created in the growing cylindrical section in proportion to their concentrations. As the system is not homogeneous, this would result in an alteration of the overall protein concentrations.

Two steps are taken to remedy the above problem. To avoid creating particles as a result of the physical growth process, a decay term was added to each state in the cylinder section of the domain, taking the form of -*growth rate · species/length of cylinder*. As a second step, the overall concentrations of MinD and MinE are maintained through homogenous production of cytoplamic MinD and MinE throughout the entire cell (see Model Formulation above).

Initial conditions were generated by setting all MinD and MinE homogeneously distributed in the *D* and *E*_*2*_ states, respectively, throughout the cell cytoplasm with cell length set equal to the starting length of the corresponding experimental kymograph. All other states were set to zero. This system was then run for 1,000 seconds without any growth to allow it to converge to a stable solution (either oscillating or stationary). The resulting distributions for each species were then taken as the initial conditions for the growing domain simulations. This procedure was justified by solving the model for a complete cell cycle (see Results) and comparing this complete simulation to individual segments used for comparison to experimental kymographs.

### Dividing Cell Simulations

To approximate the division process, we implemented a moving mesh to pinch in at the cell centre. We approximate the pinch as two semicircles with radius 0.5 *μm* (concave with respect to the cytoplasm) that are joined by a third small semicircle of 0.1 *μm* (convex with respect to the cytoplasm). At its thinnest, the radius of the cytoplasm at the pinch was 0.077 *μm*. The ratio of the expanded to constricted septum radii is 15.4% which corresponds to a ratio in the cross-sectional areas of 2.4%. This was the smallest value possible before numerical errors escalated uncontrollably. The time taken for constriction to occur was taken to be 512 *s*, as used by Sengupta [46]. As we cannot completely divide the cell in our simulations, we linearly decreased the pinching radius from the initial radius of 0.5 *μm*, to our maximum constriction at the same rate as wild type i.e. over *432* s. Once the minimum radius was reached, this shape was maintained for the remainder of the simulation. By not growing the cell during binary fission, it was possible to obtain a greater constriction of the septum, as the mesh was only being distorted in one direction rather than two.

## Results

In rod shaped *E*. *coli*, the distribution of Min proteins is effectively only a function of the position along the major axis of the cell. Thus, experimental studies have utilized kymographs to represent their data. To do this the fluorescence from a cell is collapsed to a line running along the major axis of the cell by integrating the signal perpendicular to it. This one-dimensional line is then graphed against time to create a kymograph.

**Fig 2:**
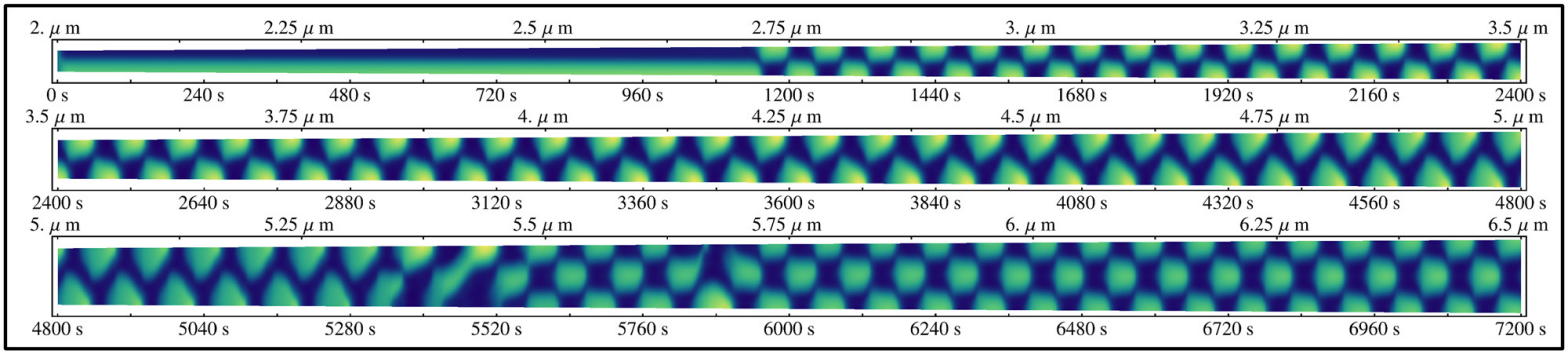
A Single Continuous Simulation of the Fully-labelled System Growing from 2 to 8 *μm* over the Course of 7200 *s*. In this and subsequent kymographs, high MinD is coloured in yellow while low MinD concentration is dark blue.

**Fig 3:**
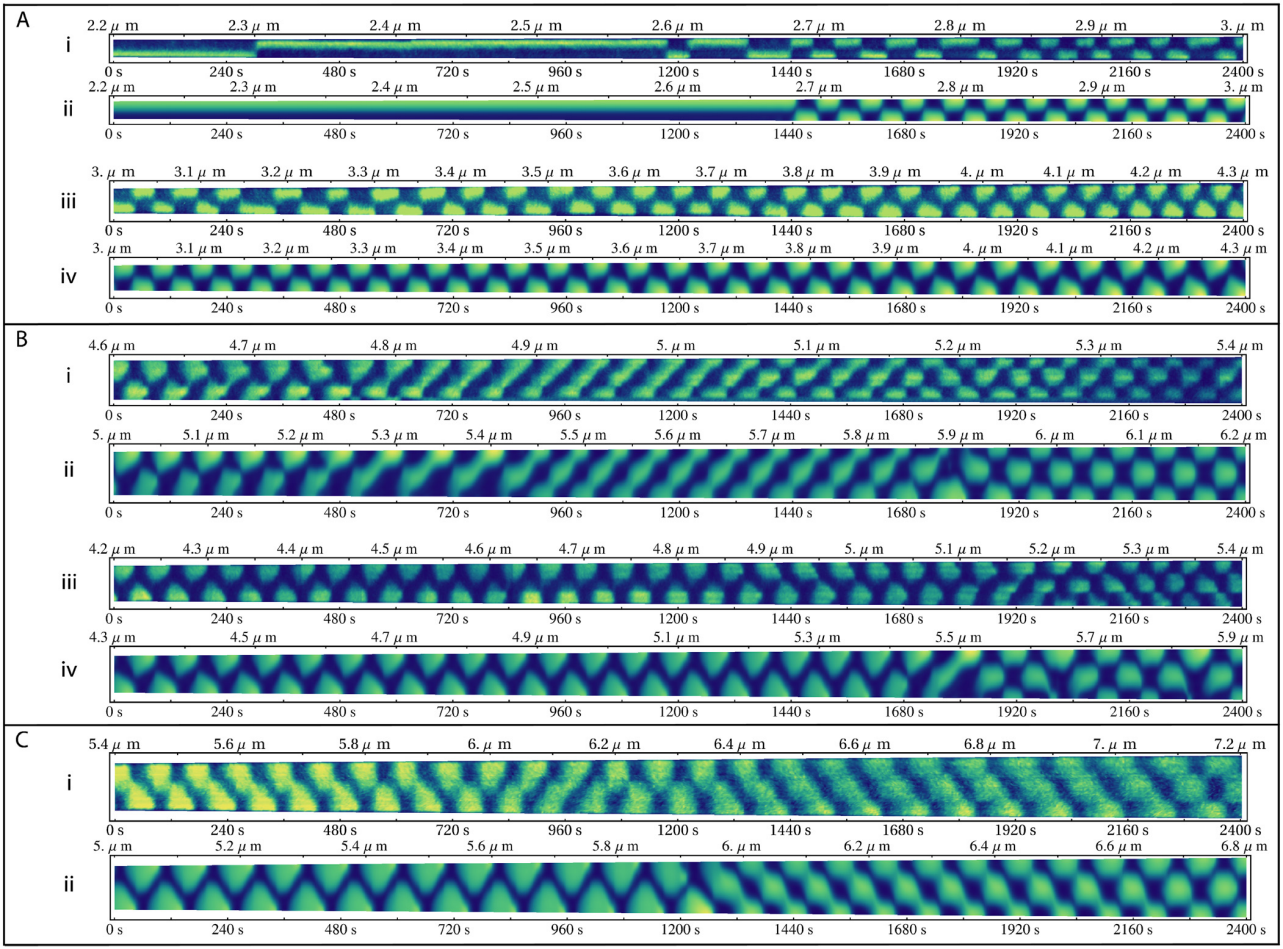
Comparison of Fully-labelled Experiments with Simulations. (A) Direct comparison between simulations and typical experimental kymographs. (i) A typical short experimental cell. Stationary patterning with stochastic switching is seen below 2.7*μm* and regular oscillations above 2.7*μm*. (ii) A simulation of a short cell with the same length and growth rate as the experimental kymograph in (i). Below 2.7*μm* it is stationary, and above it oscillates regularly. (iii) A typical mid length experimental cell showing the gradual transition from box to spearhead shaped waveforms. (iv) A simulation of a mid range cell with the same length and growth rate as the experimental kymograph in (iii). (B) The variation in the second order transition. (i) An experimental example of a first type transition with a complex mixing of first and second order modes occurring between the first and second order breather modes. (ii) A theoretical example of a first type transition with mode mixing. This cell was grown at a rate of 0.5 *nm s* ^*-1*^(iii) An experimental example displaying a second type transition moving straight from the first to the second order breather modes. (iv) A theoretical example of a second type transition grown at 0.67 *nm s* ^*-1*^. (C) Changes to Min system under a reduced MinE to MinD ratio. (i) An experimental cell showing an increase in both the period and the transition length. MinD antinodes reach closer to the centre of the cell than normal (ii) A simulation containing 90% of the usual MinE concentration displaying similar characteristics.

**Fig 4:**
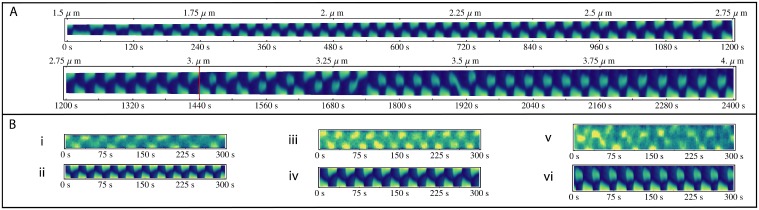
Comparison of Partially-labelled Experiments with Simulations. Note that both length and time scales have been increased (1.5 x and 2x, respectively) compared to the previous Figures so as to increase visibility. (A) A single continuous simulation of the partially-labelled system grown from 1.5 to 4 *μm* over the course of 2400 *s*. The experimentally reported length where midcell antinodes occur (3 *μm*) is marked with a red line. (B) Direct comparisons with partially-labelled experiments. (i) An experimentally measured partially-labelled cell of 1.8 *μm*. (ii) A simulation of the partially-labelled system at 1.8 *μm* displaying the patterning seen early in the cell cycle (iii) An experimental cell of a 2.5 *μm*. (iv) An equivalent theoretical simulation of a 2.5 *μm* cell. (v) Experimental midcell antinodes at 3.25 *μm*. (vi) Simulation at 3.25 *μm* also displaying midcell antinode regime.

We present the distributions of MinD resulting from our model simulations in the form of kymographs (Figs 2, 3 and 4). These are directly compared to experimental kymographs originating from the studies of Fischer-Friedrich [6] where MinD is fully-labelled (Fig 3) and Juarez [7] where MinD is partially-labelled (Fig 4). Fully-labelled MinD results in higher quality distributions. However, as MinD labelling alters aspects of division [10], we have simulated both sets of experimental conditions by adjusting our model (see below).

Overexpression of the Min proteins was required for the collection of the fully-labelled system with low background noise [6]. Such overexpression has been shown to create artificial behaviour, in particular there is no evidence that stochastic switching is present in the wild type Min system [8, 9]. While this system is highly perturbed and unlikely to reflect how the wild type Min system looks in reality, it provides a well-measured system, which in turn allows for robust tests to the accuracy of any model.

In describing the features of the kymographs, we have used the following terms. If the distribution of MinD along the cell predominantly contains a single maximum and minimum, we denote this as a first order mode (Fig 3A). When this changes to a state where there are either two maxima or two minima, we denote this state as a second order mode (Fig 3B for t > 1920 s). Patterns that do not change with time are stationary (Fig 3Aii for t < 1400 s). When either a first or second order mode oscillates with time, we call it a first or second order breather mode, respectively (Fig 3Aiii and 3Bii for t > 1900 s, respectively). Positions along the major axis of the cell where the concentration of MinD does not change with time are called nodes while locations where the concentration changes maximally with time are denoted antinodes. Simple first order breather modes have a single node around midcell (Fig 3Ai) whereas second order breather modes have two nodes (Fig 3Bii for t > 1900 s).

### Growing Domain with Fully-Labelled MinD

To examine how the model develops throughout a cell cycle, a simulation was run for the lifetime of a cell, from the length of a short newborn cell to a filamentous cell using the parameters for fully-labelled MinD. This was started at 2 *μm* and grown all the way through to 6.5 *μm* at a growth rate of 0.625 *nm s* ^*-1*^ over 120 minutes. The resulting kymograph showing the distribution of MinD as a function of time, and subsequently cell length, is presented in Fig 2, where yellow is high MinD concentration while dark blue is low (see S1 Movie).

This simulation has several defining characteristics. There is a transition from stationary to oscillating MinD distribution at approximately 2.7 *μm*. As the simulation continues past this critical point, there is a gradual transition in waveform from a rectangular shape (2.75–3.25 *μm*) to a triangular shape (4.5–5.25 *μm*). This continues until the cell length reaches approximately 5.4 *μm*, where a distinct pattern appears with MinD alternately occupying the poles and the midcell position, forming a second order breather mode (Fig 2).

### Transition From Stationary to Oscillating Pattern Arises Naturally From Model Dynamics

To evaluate the accuracy of this model for the fully-labelled MinD system, representative experimental kymographs from the work of Fischer-Friedrich [6] were selected. These kymographs do not span a complete cell division cycle due to experimental limitations. Theoretical simulations were then run between the same starting and finishing lengths as the experimental kymographs and over the same time period to provide a direct comparison. These are shown in Fig 3A (experimental kymographs in 3Ai and 3Aiii and corresponding simulations in 3Aii and 3Aiv, respectively).

Many of the physical characteristics of the Min system as seen in experimental kymographs are present in the theoretical model. Fig 3Ai illustrates how the MinD distribution in the experimental kymograph is essentially stationary at lengths less than 2.7*μm*, with MinD residing at one pole, with the phase of this first order mode switching stochastically, abruptly moving MinD to the opposite pole (Fig 3Ai at 250 *s* and twice around 1200 *s*). The equivalent theoretical simulation (Fig 3Aii) contains no stochastic terms (other than noise due to discrete computation). Hence, there is no mechanism to give rise to phase switching while the pattern is in a stationary mode i.e. MinD remains localized at the top pole from the start of the simulation continuously through to 1440 *s* (Fig 3Aii).

Once the cell grew past a critical length (2.7 *μm* in Fig 3Ai and 3Aii), both experimental (Fig 3Ai) and theoretical (Fig 3Aii) kymographs started to oscillate. As the cell elongated further, the experimental oscillations become more regular, and the agreement in period between experiment and theory improves (Fig 3A).

In cells just long enough to oscillate (2.75–3.25 *μm*) both experimental (Fig 3Ai) and theoretical (Fig 3Aii) kymographs displayed rectangular waveforms. As the cells grew, the waveform gradually changed from square wave to a more triangular shape (see the experimental and theoretical kymographs in Fig 3Aiii and 3Aiv, respectively). In both theoretical and experimental kymographs, this transition occurred by initial lengthening of the leading edge of the rectangle for cell lengths between 3.2 and 3.8 *μm* before the peak moves to the centre of the wave.

While these features are unlikely to exist in the wild type system [8, 9], they do represent a set of critical phenomena that any accurate model should be able to recreate after accounting for the fully-labelled conditions.

### Variations in Transition to the Second Order Mode in Filamentous Cells

Typically, shortly after the end of the kymograph in Fig 3Aiii cell division occurs for the fully-labelled MinD system. This creates two daughter cells in which the Min patterning returns back to the regime seen in Fig 3Ai [6]. However, occasionally a cell did not divide when it reached ~5 *μm* and continued to grow past lengths normally seen, known as filamentous growth [2].

As filamentous cells continued to grow, eventually the first order breather mode gave way to a second order breather mode. In this regime, MinD oscillated between localizing simultaneously at both poles before localizing to the centre of the cell. Examples of second order breather modes are seen in every kymograph in Fig 3B after 2000 *s* and in Fig 3C.

Visual inspection of the kymographs of filamentous cells provided by Fischer-Friedrich for the fully-labelled MinD system suggested that there are two broad classes of transition from the first to the second order breather modes. The first type of transition contained an intermediary travelling wave pattern (diagonal stripes in Fig 3Bi & 3Bii between 600 and 1400 *s*). This transitional pattern appeared to be a superposition of a first and second order breather mode. The travelling wave was moving from the bottom of the cell to the top, giving rise to the striped kymograph. After further cell growth, the first order mode decayed, leaving a simple second order breather mode (Fig 3Bi & 3Bii at times > 1800 *s*).

In contrast, the second type of transition skipped this travelling wave phase and went straight from a first to a second order breather mode. An example of this type of transition is shown in Fig 3Biii where the first order breather mode was maintained all the way to 1900 seconds before a sudden transition to a stable second order breather mode occurs.

By varying the starting length and growth rate of our simulations, we were able to replicate both of these types of transition types. These are shown in Fig 3Bii and 3Biv respectively. We note that the transition length of the model, approximately 5.5 *μm*, differs from that seen in the experimental kymographs (Fig 3Bi and 3Biii) however, it was within the range seen experimentally (approximately 4.8–7.2 *μm*).

### Min Patterning is Dependent on the MinE:MinD Ratio

To test the robustness of the model to variations in the relative concentrations of MinD and MinE, a simulation was run with 90% of the wild type MinE concentration (Fig 3Cii). Although it shows similar features to wild type simulations, distinct differences are evident. The reduction of MinE concentration resulted in an increase in the period of oscillation compared to the wild type kymographs (approximately 100 *s* versus 80 *s*) which was accompanied by a proportional increase in width of the waveforms as a function of time. This is in qualitative agreement with previous stochastic [26] and deterministic [24, 30] models that have also shown an increase in period with a decrease in MinD:MinE ratio. Unfortunately, to date, there is no quantitative data relating protein ratios to the period of oscillation [30].

Transitions to the second order breather mode occur at a cell length of 6 *μm* compared to 5.5 *μm* for wild type MinE:MinD ratios. The triangular waveforms of high MinD concentration interdigitate (Fig 3Cii, t < 1200s), indicating that high MinD concentrations are seen all the way from the cell pole to beyond the cell midline. High MinD concentration crossing the cell midline was also seen in some simulations with wild type MinE:MinD ratios (Fig 3Biv near 1500 s), however, the extent to which MinD crossed the midline was smaller. The extent to which MinD crosses the cell midline increases as the MinE:MinD ratio was decreased until eventually the waveforms originating at each pole begin to merge (see S2 Text for details).

Within the set of experimental data with fully-labelled MinD [6], one distinct experimental kymograph displayed the same characteristics as the simulation with reduced MinE concentration (Fig 3Ci). Comparing this to the kymographs in Fig 3Bi and 3Biii, one sees that the period has increased. Also, in the first part of the kymograph where the first order breather mode dominates the pattern, the regions of high MinD concentration extend past cell midline and partially merge as per the simulation. The transition to the second order breather mode in the experimental kymograph occurs at a cell length of approximately 6.4 *μm* which was longer than the median length for transitions seen in the experimental set.

### Min Oscillations with Partially-Labelled MinD

Touhami demonstrated that there is a large discrepancy in the period of oscillation between the fully and partially-GFP-labelled Min systems [10]. The difference in period reported is much larger than the reported changes in the period of oscillation of the Min system throughout the cell cycle [6]. As different base strains were used for the different types of labelling experiments, there are many potential sources for the discrepancy in oscillation period.

It has been proposed that the appearance of the stationary phase in short cells as observed by Fischer-Friedrich is a result of over expression of the Min system [9]. If Min protein concentration is the sole source of differences between the experimental results for fully-labelled versus partially-labelled cells, we should be able to model partial-labelling by reducing Min protein concentration. While we find that reducing the concentration of the Min system is sufficient to abolish this stationary phase of patterning and decrease the period of oscillation, it is not capable recreating the partially-labelled data of Juarez [7]. A kymograph of the reduced concentration system is shown in S1 Fig. In particular, the transition from first to second order mode remains longer than observed experimentally in partially-labelled cells. In the fully-labelled Fischer-Friedrich experiment this transition occurs between 4.7 and 5.5 *μm* (Fig 2), while in the Juarez experiment the transition to mid cell antinodes occurs at 3 *μm* (Fig 4). This transition to mid cell antinode patterning has been independently observed by fluorescently labelling MinC [45]. Using our model that fits the fully-labelled kymographs and reducing the Min protein concentration the transition occurs at 4.5 *μm* (S1 Fig) which is not consistent with the partially-labelled experimental kymographs. Furthermore, there is only a minimal region where mid cell antinodes are observed in this model (S1 Fig from 4000 to 5000 s), again differing from experiment. Thus, a reduction in Min protein concentration alone is not sufficient to allow our model to fit both fully- and partially-labelled experimental data.

Plotting the change in period due to perturbations in each parameter in our model showed that the ATPase/heterotetramer dissociation parameter, *ω*_*hydr*_, had the greatest influence on the period of oscillation (see S3 Text for details). It has recently been shown experimentally that GFP tagging can cause artefactual aggregation of native homomultimeric proteins [47]. If GFP were having a stabilizing effect on the membrane-bound MinD-GFP dimer then we would expect that GFP labelling would decrease the rate of monomerisation, which, in our model, is a component of the ATPase and MinDE heterotetramer dissociation reaction modelled by the rate constant *ω*_*hydr*_. Two approaches to avoid GFP artefacts have been the use of GFP variants that prevent GFP self-association [47], and minimally labelling proteins with GFP *in vivo* [48].

Combined, these results indicate that, as a first approximation, altering this parameter (*ω*_*hydr*_) could counteract the effects of GFP-labelling on the function of MinD. Rescaling this rate from 0.12 *s* ^-1^ for fully-labelled MinD to 0.5 *s* ^-1^ for the partially-labelled system resulted in a decrease in the oscillation period so that simulations matched experimental data (Fig 4). In addition, GFP labelling alters the diffusion coefficient of MinD in solution; hence it was increased from 16 to 24 *μm* ^2^ *s* ^-1^. We note that changing the diffusion constant had a minimal effect on simulations (see S4 Text for comparison).

Results of a simulation with this modified set of parameters are shown in Fig 4A (see S2 Movie). In this simulation, the cell was grown at a rate of 1.05 *nm s* ^*-1*^ which is faster than the growth rates seen in the fully-labelled system but correlates to a biomass doubling time of approximately 30 minutes. This was chosen to be consistent with *E*. *coli* in rich media. In any case, changes in growth rate have a negligible effect on transition lengths and patterning within stable patterning regions. This can be seen by comparing the theoretical kymographs in Fig 4B where there was no cell growth, with the corresponding lengths in Fig 4A. Many of the characteristics typically associated with the partially-labelled Min system are seen in this simulation (Fig 4A).

Using these parameters, the length at which the cell transitioned from a stationary to an oscillating MinD pattern occurred at less than 1.5 *μm*, which is below the minimum length of typical *E*. *coli* cells. From 1.5 *μm* through to 3 *μm* the model predicts that partially-labelled MinD rapidly oscillates from pole to pole (Fig 4A). MinD resides close to one of the cell poles giving rise to a definite bare zone at the midcell. A direct comparison between a short cell of 1.8 *μ*m from Juarez’ experiments [7] and the model is made in Fig 4Bi and 4Bii, respectively. As partial labelling provides a much weaker signal compared to the fully-labelled system, it is difficult to characterize the experimental waveform. However, the antinodes of both experimental and theoretical kymographs extend a similar distance from the poles of the cell and have a similar period. Such characteristics continue as the cell grows, as demonstrated for a 2.5 *μ*m cell (Fig 4Biii and 4Biv).

Experiments on cells with partially-labelled MinD show a transition to a different type of patterning at 3 *μm* [7]. This length is marked on the second line of the model kymograph in Fig 4A by a red line. This regime is dominated by patterning where the high concentration of MinD at one end of the cell moves away from the pole to a region adjacent to the cell midline. This type of patterning was previously coined “midcell pausing” [7], however, we feel that this is misleading as the word “pausing” suggests a change in the time dependent behaviour of the Min proteins. To avoid this, we refer to this phenomenon as “midcell antinodes” as this accurately describes an increase in MinD concentration in the midcell region that arises from the dynamics of the Min system.

Midcell antinodes are seen in both experiments and simulations (Fig 4Bv and 4Bvi). Our simulation for a full cell growth demonstrates a strong tendency for anitinode formation at midcell to favour one end of the cell with only occasional switching to the opposite pole (Fig 4A. cell length > 3 *μm*). This is consistent with qualitative experimental reports [7]. Such switching indicates that there is an instability underlying this behaviour with the switching in the model likely being triggered by numerical imprecision in the computation.

### Temperature Dependence of Period of Oscillation

It has been suggested that ATP hydrolysis is the rate-limiting step in the Min cycle [24]. The ability for changes to the rate of ATP hydrolysis to account for changes in the period of oscillation have also been explored in similar models [30]. If this is correct for our model, we expect that the temperature dependence of the system could be modelled by changing the rate of ATP hydrolysis. The model implicitly contains the ATP hydrolysis step within the parameter *ω*_*hydr*_ which also includes heterotetramer dissociation (see Model Formulation for details). A Boltzmann factor was subsequently applied to this term. The Boltzmann factor takes the form $\text{exp}\left(\frac{\varepsilon \left(T-T_{0}\right)}{kTT_{0}}\right)$ where *ε* is the activation energy for that the reaction that includes ATP hydrolysis and tetramer dissociation, *T*_*0*_ is the temperature under which the experiments of Juarez were conducted (32°C) [7]) and *k* is Boltzmann’s constant.

**Fig 5:**
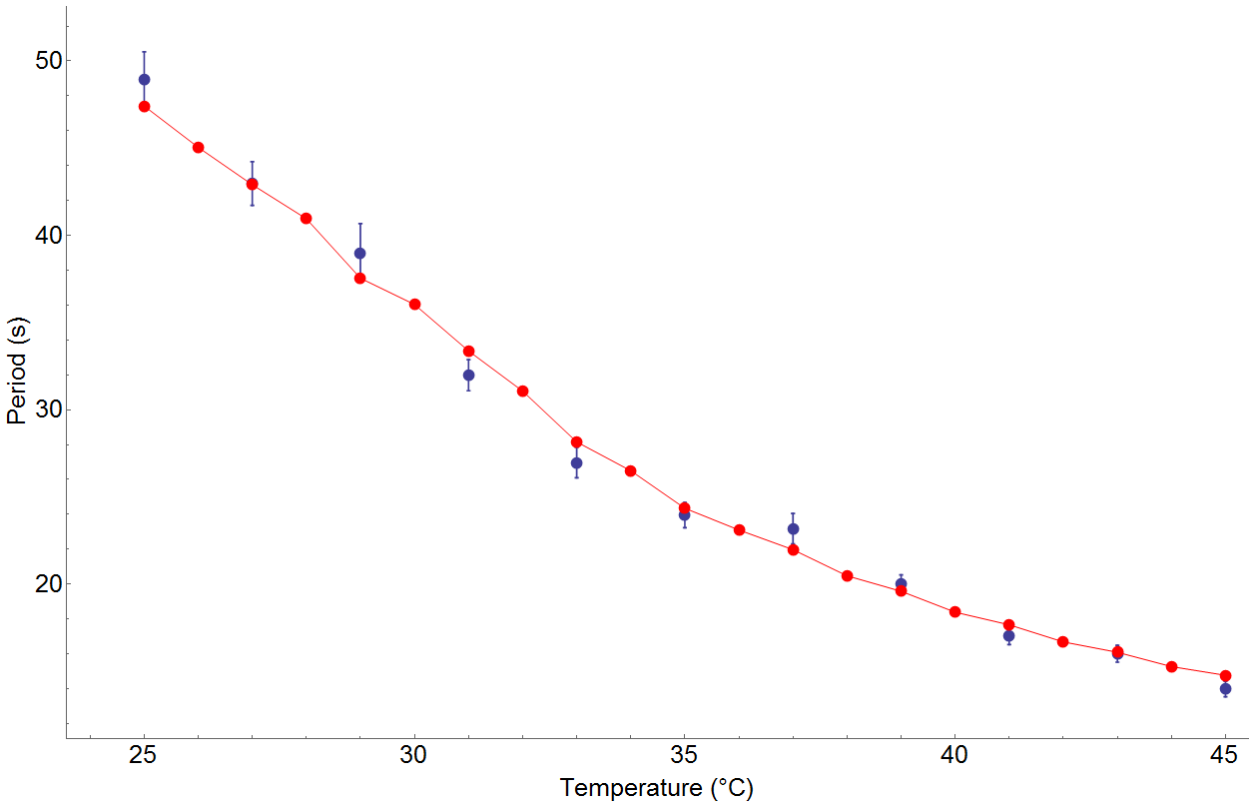
Temperature Dependence of the Period of Oscillation for the Min Protein System. Experimental data with error bars is shown in blue [49]. The periods extracted from individual simulations at each temperature are shown in red with straight lines joining simulation points.

The resulting relationship between temperature and MinD oscillation period of the model is shown in red in Fig 5. Close examination reveals that the model results fit the experimental data for the dependence of oscillation period on temperature [49] with a standard deviation of 1.03 s between model (Fig 5, red) and experimental (Fig 5, blue) data points. The activation energy for the best fit was equal to 11.5 kcal/mol, which coincidentally is approximately equal to the free energy of ATP hydrolysis.

### Simulating Min Patterning During Cell Division

Juarez measured the changes in Min dynamics during cell division (using partially-labelled MinD) to investigate the mechanism behind the cell’s ability to equally partition the Min proteins between daughter cells [7]. It was shown that, in constricting cells, patterning dominated by antinodes forming at midcell seen in Fig 4A soon gives way to regular, second order oscillation around the time of septum closure. This second order breather mode is symmetric, so on average both MinD and MinE would be divided equally between each cell half throughout the oscillation cycle and hence partition equally to each daughter cell. A similar process has also been reported to occur in cells where MinD is fully labelled, with Min patterning continues unimpeded for both daughter cells following cell division [6].

**Fig 6:**
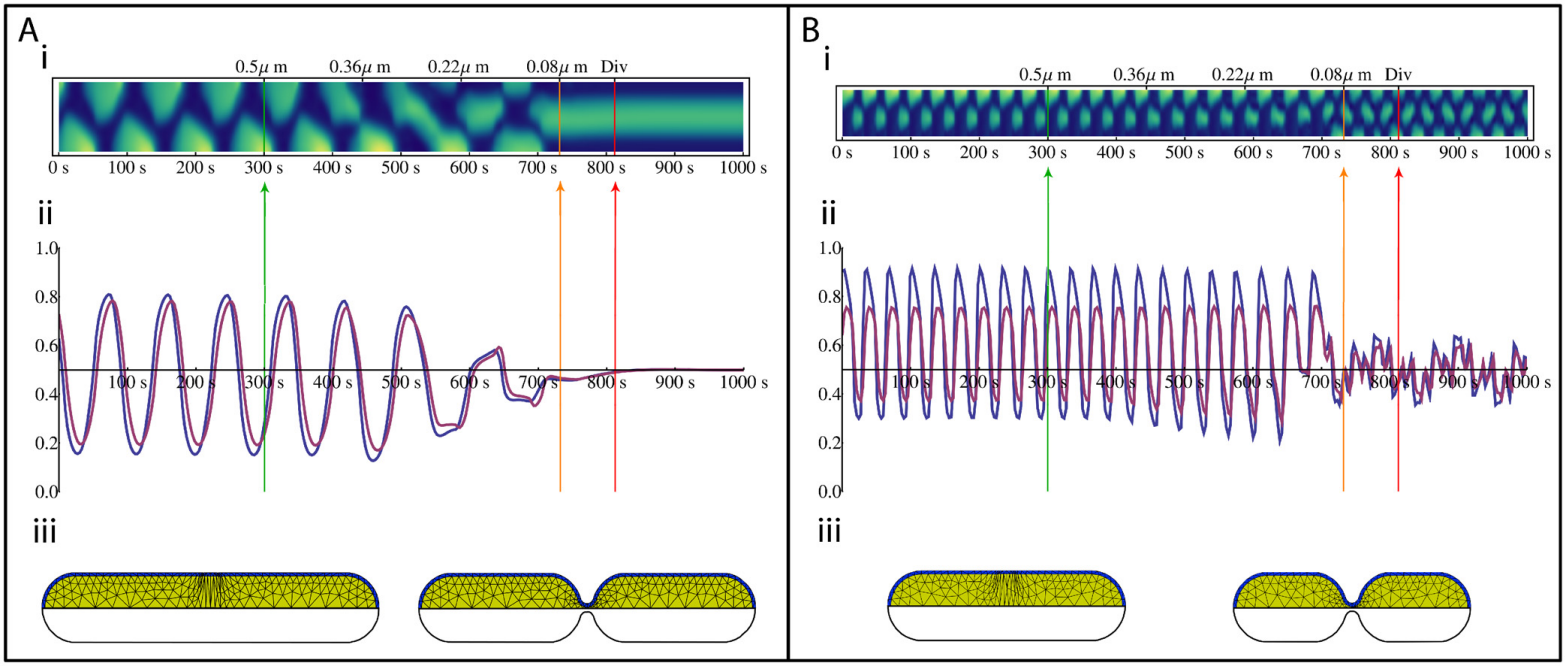
Simulation of Cell Division. Septum constriction begins after 300 *s* and is halted after 732 *s*. If constriction continued, cytokinesis would occur at 812 *s*. (A) Fully-labelled system run at 5 *μm*. (B) Partially-labelled system run at 3.5 *μm*. (i) The kymograph of the MinD distribution over the division process. Lengths on top of the kymograph denote the minimum radius of the septum at each point in time. (ii) The proportion of MinD (blue) and MinE (purple) in the future top daughter cell. The green and orange vertical lines mark the beginning and end of constriction respectively. The red vertical line marks the theoretical binary fission point. (iii) The cell geometry before (left) and after (right) constriction.

To simulate the cell division process in both the fully-labelled and partially-labelled systems, the model equations were solved for a fixed cell length while the midcell region was continuously constricted (see Methods; Fig 6). This builds on the work of Di Ventura who solved a model of the Min system for various static constriction radii and showed that it was sufficient to induce a transition to a second order mode [8]. To be consistent with Juarez’s observation of high MinD concentration near the cell midline prior to cell division, we performed the partially-labelled study at a cell length 3.5 *μm*, where the system is firmly in the regime supporting midcell antinodes (Fig 4A). The fully-labelled system was modelled with a 5 *μm* cell, which is within the range where cell division is observed. However, from our simulations, a fully-labelled 5 *μm* cell shows a stable first order breather mode (Figs 2, 3B and 3C) which will continue indefinitely in the absence of septum constriction.

Results of the simulations of the fully-labelled and partially-labelled systems are shown in Fig 6A and 6B, respectively (see S3 and S4 Movies, respectively). For the fully-labelled cell prior to the initiation of division (t < 300 s, green line in Fig 6A), the proportion of Min proteins in the top cell half varies from approximately 13% to 81% for MinD and 17% to 78% for MinE across a single period (Fig 6Aii). With the onset of septum constriction, oscillations are initially unaffected as the septum radius is reduced (Fig 6Ai and 6Aii). Once the radius of the septum has reduced to approximately 0.25 *μm*, half its initial value, we see a critical point, following which an erratic transition period ensues. Eventually this stabilises into a stationary second order mode where MinD is symmetrically localized to the centre of the cell. After 432 seconds of constriction (732 seconds of simulation), when the septum has reached the maximum constriction possible, indicated in the figure with an orange line, the proportion of MinD and MinE in the top cell is within 4% of parity, following which it then continues to converge towards equal distribution. At this stage, oscillations cease and the MinD distributions adopts a stationary second order mode (Fig 6Ai with t > 750 *s*).

Both MinD and MinE asymptotically approach symmetric distribution in the two halves of the cell from the same side, such that even if their individual partitioning has not completely converged to 50%, as is the case in this simulation, their stoichiometric ratio is approximately constant. Consequently this increases the stability of the patterning in the daughter cells, as the stability of future patterning is more susceptible to differences in the MinE to MinD ratio than their absolute concentrations [11]. The root mean square of the difference between the division ratios of MinD and MinE is 0.35%,

A model calculation for cell division in the partially-labelled system is shown in Fig 6B. As seen in Fig 6Bii partially-labelled cell leads to a much greater asymmetry in the distribution of Min proteins in each half of the cell when compared to the fully-labelled system (Fig 6Aii), with MinD varying from 30% to 90% and MinE from 37% to 76% in the top half of the cell. As per the fully-labelled system, the oscillations are initially unaffected by the divisome constriction. However, as constriction continues, the distribution of Min proteins in the two halves of the cell becomes more symmetric over an oscillation period. Fig 6Bii shows this clearly around 600 seconds where the percentages oscillate from 20% and 90% for MinD and 28% and 76% for MinE.

At approximately 700 s, a transition to a second order breather mode occurs. As seen in the kymograph in Fig 6Bi, this second order mode is not completely symmetric which leads to the small scale fluctuations in Min protein distributions (seen after 700 seconds in Fig 6Bii). Between the time that the constriction is halted due to numerical constraints (shown by the orange line) and when binary fission is likely to occur (denoted by the red line), the root mean square of the difference between parity and the percentage of Min protein in both halves was 7.7% and 6.1% for MinD and MinE respectively. These results are consistent with the experimental measurements of the distribution of MinD between daughter cells [7]. Similar to the fully-labelled model, MinE closely follows the MinD oscillations, so the root mean square of the difference between the MinD and MinE percentages is 4.75%, which is less than the absolute fluctuation.

## Discussion

We have produced a model of the Min protein system based on experimentally determined molecular interactions. This model reproduces the main characteristics of the *in vivo* Min system for a full cell cycle using a single set of parameters with only the rate constant controlling MinD ATPase and *d*_*2*_ *e*_*2*_ heterotetramer dissociation (*ω*_*hydr*_) being varied to account for GFP labelling or the effects of temperature. Comparison of experimental and model kymographs showing the distribution of MinD as a function of time and cell length suggests that the model accounts for the major interactions in the Min system that are responsible for MinD spatiotemporal patterning.

The model naturally accounts for the major transitions of the Min protein system during cell growth and division. In particular, the model reproduces the gradual changes in appearance of MinD waveforms in the kymographs as a function of time (from square wave to triangular waveforms). This suggests that the dominant interactions of the physical system have been successfully incorporated into the model so as to give rise to the same non-linearities that are responsible for patterning.

Critical transitions in MinD patterning as a function of cell length for cells with both fully-labelled and partially-labelled MinD-GFP were reproduced by the model. For the fully-labelled cells, the model reproduces the transition from stationary patterning to MinD oscillations at 2.75 *μm* and, in filamentous cells, the various MinD patterns observed in transitions from the first to the second order breather modes. While these patterning phenomena are unlikely to exist in wild-type cells [8, 9], the ability to recreate them is an important check for the model’s validity.

The model reproduces Min dynamics for partially-labelled cells by increasing the rate constant controlling MinD ATPase and heterotetramer dissociation by a factor of approximately four. Under these conditions, the MinD pattern is always oscillating for realistic cell lengths (i.e. no cells have stationary MinD distributions), explaining the discrepancies between experiments on fully-labelled [6] and partially-labelled [7] cells.

The model also reproduces the development of antinodes near the septum in cells approaching cell division, previously termed midcell pausing, which is observed in partially-labelled cells [7] but not in fully-labelled cells [6]. Our findings show that midcell antinodes can happen autonomously via the known interactions amongst MinD, MinE and the membrane, and would not require changes in membrane curvature or other molecules located at midcell. It seems likely that midcell antinodes may be an intrinsic feature of Min patterning that aids in the equal partitioning of Min proteins into daughter cells by signalling when the Min system is ready to transition to a second order mode synchronously with septum closure. The slight filamentation of the fully labelled system that lacks midcell antinodes suggests that the partially-labelled system may be more reflective of oscillations of unlabelled proteins.

Consistent with reports that MinD ATPase is the rate limiting step of the Min cycle [30], the temperature dependence of the Min oscillation period can be reproduced by this model by multiplying the rate constant controlling MinD ATPase by a Boltzmann factor with the activation approximately energy equal to the free energy of ATP hydrolysis.

A potential molecular mechanism that may underlie the differences between the fully and partially-labelled systems is the ability of GFP to self-interact and stabilize protein complexes and cause aggregation [47]. If this is the case for MinD, the extent to which it occurs will be primarily dependent on the variant of the fluorescent protein used, and the relative amount of the MinD-GFP fusion protein to wild type MinD in the cell. This may potentially explain why Di Ventura *et al*. were unable to reproduce the stationary patterning of Fischer Friedrich in a fully GFP labelled MinD system, nor the midcell antinodes of Juarez in a partially-labelled system [8].

A striking technical advancement of the model is its ability to partition MinD and MinE into two daughter cells nearly equally and with essentially the same MinE:MinD ratio as the parent cell, in a single continuous simulation. This partitioning is the result of the interplay between Min dynamics and the geometry of the dividing cell. Prior to midcell constriction, the distribution of MinD is determined by a mixture of first and second order breather modes and is clearly asymmetric in terms of the Min protein distribution in the future daughter cells. The constriction of the septum facilitates the decay of the first order mode and stabilizes the symmetric second order mode. The symmetry of this mode is responsible for the equipartitioning of both MinE and MinD.

The existence of Min polymers (*in vitro* and *in vivo*) and their relevance for Min patterning and cell division remains controversial [21, 50–53]. No polymers or complex structures have been incorporated into our model. It is possible that polymer formation, or some other complex besides dimerisation, contributes to the stability of membrane-bound MinD dimers. Our model does not require anisotropic diffusion of MinD or curvature sensing in MinD membrane binding in order to produce patterning. Although curvature sensing could contribute to MinD binding to the end cap in *E*. *coli* cells, it does not account for the MinD patterns observed in either filamentous cells [54, 55] or in the artificial planar bilayer experiments [12]. While all of these reactions may be present and may play a role in optimizing the performance of the Min system, this work demonstrates that they are unnecessary to produce the main characteristics of the Min system as seen *in vivo*.

It has recently been reported that MinD forms co-polymers with MinC [56]. The deletion of MinC has been shown to have little effect on patterning aside from a slight change in period [5]. As a result, it is probable that these polymers aid in the function of MinC inhibiting FtsZ rather than aiding the patterning of the Min system. Indirect evidence that MinD binds to DNA [57] also suggests that the inhibition mechanism of FtsZ polymerization by the Min system may be far more complicated than currently assumed.

We have modelled the system using partial differential equations. Whether a stochastic variant would be able to account for stochastic switching in the fully labelled system for short cells remains an open question.

Our molecular model provides a single mathematical description that can account for the observed patterning under diverse experimental conditions. Thus, it provides a basis for unifying our understanding of the Min system and its role in cell division. The Min system has been studied and modelled in a plethora of different phenotypes. What proportion of these phenotypes can be explained by a single model with a single set of parameters will have to await further investigation.

## Supporting Information

S1 Fig Fully-labelled System with Reduced Protein Concentration. The fully-labelled system was simulated with MinD and MinE each containing a third of their normal concentrations (463 *μm* ^-3^ (0.76 *μM*) and 162 *μm* ^-3^ (0.26 *μM*), respectively). The resulting kymograph displays no stochastic switching at short cell lengths, with the Min system oscillating from the start of the simulation with a period of approximately 38 s. The system begins to transition away from a pure first order mode at approximately 4000s (with a length of 4.5 *μm*). This transitional segment contains mid cell antinodes before the second order mode dominates at 5000s (cell length of 5.1 *μm*).
(TIFF)

S2 Fig Comparison Between Variants of the Min Model. (A) Kymograph of the model used for the results in this paper, the reactions for which are summarised in Fig 1. B) A model with the same basic reactions as (A) except that MinE binding to the membrane is mediated by MinD dimers. That is, MinE in solution (*E*_*2*_) binds with membrane-bound MinD dimers (*d*_*2*_) to form a heterotetramer (*d*_*2*_ *e*_*2*_) instead of binding directly to the membrane.
(TIF)

S3 Fig Breakdown of the Min Patterning Under Reduced MinE. The resulting kymographs from simulations run with (A) 90% (B) 85% (C) 80% (D) 75% (E) 70% of the wild type concentration of MinE.
(TIF)

S4 Fig Correlations Between Individual Parameter Variation and Change in Period of the Min Oscillation. Each parameter is scaled from 0.9 to 1.1 times its original value while the remaining parameters are held constant. The period is then determined by taking the maximum Fourier component of each resulting simulation.
(TIF)

S5 Fig Comparison of MinD and MinD-GFP Diffusion Constants. (A) Kymograph of the system with the diffusion of MinD in solution set to 24 *μm* ^2^ *s* ^-1^ (B) Kymograph grown over the same length range with the same parameters except that the MinD in solution is set to the experimental measurement for MinD-GFP of 16 *μm* ^2^ *s* ^-1^ [41]. The experimentally reported length where midcell antinodes occurs (3 *μm*) is marked with a red line.
(TIF)

S1 Movie An Animation of a Simulation for the Fully-labelled Min System. Key features of this simulation include the transition from stationary to oscillating patterning after 18 seconds of animation when the cell reaches 2.7 *μm* and the transition to the second order mode at 1:30. In this animation, one second of movie corresponds to 20 seconds of real time. This movie represents the same data displayed as a kymograph in Fig 2.
(AVI)

S2 Movie An Animation of the Partially-labelled Min System. The key feature of this simulation is the onset of midcell antinodes after 48 seconds. In this animation, one second of movie corresponds to 20 seconds of real time. This movie represents the same data displayed as a kymograph in Fig 4A.
(AVI)

S3 Movie An Animation of the Cell Division Process for the Fully-labelled Min System. After 5 seconds of movie time, constriction begins, and proceeds through until maximally constricted at approximately 12 seconds. If constriction were to continue, complete dell division would occur at 13 seconds. In this animation, one second of movie corresponds to approximately one minute of real time. This movie represents the same data displayed as in Fig 6A.
(AVI)

S4 Movie An Animation of the Cell Division Process for the Partially-labelled Min System. After 11 seconds of movie time, constriction begins, and proceeds through until maximally constricted at approximately 22 seconds. If constriction were to continue, complete dell division would occur at 24 seconds. In this animation, one second of movie corresponds to approximately 30 seconds of real time. This movie represents the same data displayed as in Fig 6B.
(AVI)

S1 Table Rate Parameters for Final Model Compared to MinD Mediated MinE Binding Model. (DOC)

S1 Text MinD Mediated MinE Binding Alternative Model. (DOC)

S2 Text Breakdown of Min Patterning due to Decreasing MinE:MinD Ratio. (DOCX)

S3 Text Variation of Oscillation Period Due to Parameter Perturbation. (DOC)

S4 Text Impact of Diffusion Rescaling. (DOCX)

## References

[1] Yu XC, Margolin W. FtsZ ring clusters in min and partition mutants: role of both the Min system and the nucleoid in regulating FtsZ ring localization. Mol Microbiol. 1999;32(2):315–26. 10231488 10.1046/j.1365-2958.1999.01351.x

[2] Bi E, Lutkenhaus J. Cell division inhibitors SulA and MinCD prevent formation of the FtsZ ring. J Bacteriol. 1993;175(4):1118–25. 8432706 10.1128/jb.175.4.1118-1125.1993 PMC193028

[3] Oliferenko S, Chew TG, Balasubramanian MK. Positioning cytokinesis. Genes Dev. 2009;23(6):660–74. 10.1101/gad.1772009 19299557

[4] de Boer PA, Crossley RE, Rothfield LI. A division inhibitor and a topological specificity factor coded for by the minicell locus determine proper placement of the division septum in E. coli. Cell. 1989;56(4):641–9. 2645057 10.1016/0092-8674(89)90586-2

[5] Raskin DM, de Boer PA. Rapid pole-to-pole oscillation of a protein required for directing division to the middle of Escherichia coli. Proc Natl Acad Sci U S A. 1999;96(9):4971–6. 10220403 10.1073/pnas.96.9.4971 PMC21801

[6] Fischer-Friedrich E, Meacci G, Lutkenhaus J, Chaté H, Kruse K. Intra- and intercellular fluctuations in Min-protein dynamics decrease with cell length. Proc Natl Acad Sci U S A. 2010;107(14):6134–9. 10.1073/pnas.0911708107 20308588 PMC2851992

[7] Juarez JR, Margolin W. Changes in the Min oscillation pattern before and after cell birth. J Bacteriol. 2010;192(16):4134–42. 10.1128/JB.00364-10 20543068 PMC2916429

[8] Di Ventura B, Sourjik V. Self-organized partitioning of dynamically localized proteins in bacterial cell division. Mol Syst Biol. 2011;7:457 10.1038/msb.2010.111 21206490 PMC3049411

[9] Sliusarenko O, Heinritz J, Emonet T, Jacobs-Wagner C. High-throughput, subpixel precision analysis of bacterial morphogenesis and intracellular spatio-temporal dynamics. Mol Microbiol. 2011;80(3):612–27. 10.1111/j.1365-2958.2011.07579.x 21414037 PMC3090749

[10] Touhami A, Jericho M, Rutenberg AD. Temperature dependence of MinD oscillation in Escherichia coli: running hot and fast. J Bacteriol. 2006;188(21):7661–7. 10.1128/JB.00911-06 16936014 PMC1636269

[11] Pichoff S, Vollrath B, Touriol C, Bouche JP. Deletion analysis of gene minE which encodes the topological specificity factor of cell division in Escherichia coli. Mol Microbiol. 1995;18(2):321–9. . 8709851 10.1111/j.1365-2958.1995.mmi_18020321.x

[12] Loose M, Fischer-Friedrich E, Ries J, Kruse K, Schwille P. Spatial regulators for bacterial cell division self-organize into surface waves in vitro. Science. 2008;320(5877):789–92. 10.1126/science.1154413 18467587

[13] Lackner LL, Raskin DM, de Boer PAJ. ATP-dependent interactions between Escherichia coli Min proteins and the phospholipid membrane in vitro. J Bacteriol. 2003;185(3):735–49. 12533449 10.1128/JB.185.3.735-749.2003 PMC142821

[14] Wu W, Park KT, Holyoak T, Lutkenhaus J. Determination of the structure of the MinD-ATP complex reveals the orientation of MinD on the membrane and the relative location of the binding sites for MinE and MinC. Mol Microbiol. 2011;79(6):1515–28. 10.1111/j.1365-2958.2010.07536.x 21231967 PMC3077903

[15] Mileykovskaya E, Fishov I, Fu X, Corbin BD, Margolin W, Dowhan W. Effects of phospholipid composition on MinD-membrane interactions in vitro and in vivo. J Biol Chem. 2003;278(25):22193–8. 12676941 10.1074/jbc.M302603200

[16] Taghbalout A, Ma L, Rothfield L. Role of MinD-membrane association in Min protein interactions. J Bacteriol. 2006;188(8):2993–3001. 16585760 10.1128/JB.188.8.2993-3001.2006 PMC1446990

[17] Ghasriani H, Goto NK. Regulation of symmetric bacterial cell division by MinE: What is the role of conformational dynamics? Commun Integr Biol. 2011;4(1):101–3. 10.4161/cib.4.1.14162 21509194 PMC3073286

[18] Park K-T, Wu W, Battaile KP, Lovell S, Holyoak T, Lutkenhaus J. The Min oscillator uses MinD-dependent conformational changes in MinE to spatially regulate cytokinesis. Cell. 2011;146(3):396–407. 10.1016/j.cell.2011.06.042 21816275 PMC3155264

[19] Hsieh C-W, Lin T-Y, Lai H-M, Lin C-C, Hsieh T-S, Shih Y-L. Direct MinE-membrane interaction contributes to the proper localization of MinDE in E. coli. Mol Microbiol. 2010;75(2):499–512. 10.1111/j.1365-2958.2009.07006.x 20025670 PMC2814086

[20] Hu Z, Lutkenhaus J. Topological regulation of cell division in E. coli. spatiotemporal oscillation of MinD requires stimulation of its ATPase by MinE and phospholipid. Mol Cell. 2001;7(6):1337–43. 11430835 10.1016/s1097-2765(01)00273-8

[21] Loose M, Kruse K, Schwille P. Protein self-organization: lessons from the min system. Annu Rev Biophys. 2011;40:315–36. 10.1146/annurev-biophys-042910-155332 . 21545286

[22] Kruse K, Howard M, Margolin W. An experimentalist's guide to computational modelling of the Min system. Mol Microbiol. 2007;63(5):1279–84. 17302810 10.1111/j.1365-2958.2007.05607.x PMC4758205

[23] Aragon JL, Barrio RA, Woolley TE, Baker RE, Maini PK. Nonlinear effects on Turing patterns: time oscillations and chaos. Phys Rev E Stat Nonlin Soft Matter Phys. 2012;86(2 Pt 2):026201 . 23005839 10.1103/PhysRevE.86.026201

[24] Huang KC, Meir Y, Wingreen NS. Dynamic structures in Escherichia coli: spontaneous formation of MinE rings and MinD polar zones. Proc Natl Acad Sci U S A. 2003;100(22):12724–8. 14569005 10.1073/pnas.2135445100 PMC240685

[25] Hu Z, Gogol EP, Lutkenhaus J. Dynamic assembly of MinD on phospholipid vesicles regulated by ATP and MinE. Proc Natl Acad Sci U S A. 2002;99(10):6761–6. 11983867 10.1073/pnas.102059099 PMC124476

[26] Kerr RA, Levine H, Sejnowski TJ, Rappel W-J. Division accuracy in a stochastic model of Min oscillations in Escherichia coli. Proc Natl Acad Sci U S A. 2006;103(2):347–52. 16387859 10.1073/pnas.0505825102 PMC1326155

[27] Huang KC, Wingreen NS. Min-protein oscillations in round bacteria. Phys Biol. 2004;1(3–4):229–35. 16204843 10.1088/1478-3967/1/4/005

[28] Tostevin F, Howard M. A stochastic model of Min oscillations in Escherichia coli and Min protein segregation during cell division. Phys Biol. 2006;3(1):1–12. 10.1088/1478-3975/3/1/001 16582457

[29] Fange D, Elf J. Noise-induced Min phenotypes in E. coli. PLoS computational biology. 2006;2(6):e80 16846247 10.1371/journal.pcbi.0020080 PMC1484588

[30] Halatek J, Frey E. Highly Canalized MinD Transfer and MinE Sequestration Explain the Origin of Robust MinCDE-Protein Dynamics. Cell Reports. 2012;1(6):741–52. 10.1016/j.celrep.2012.04.005 22813748

[31] Bonny M, Fischer-Friedrich E, Loose M, Schwille P, Kruse K. Membrane Binding of MinE Allows for a Comprehensive Description of Min-Protein Pattern Formation. PLoS computational biology. 2013;9(12):e1003347 10.1371/journal.pcbi.1003347 24339757 PMC3854456

[32] Meacci G, Kruse K. Min-oscillations in Escherichia coli induced by interactions of membrane-bound proteins. Phys Biol. 2005;2(2):89–97. 16204861 10.1088/1478-3975/2/2/002

[33] Cahn JW, Hilliard JE. Free Energy of a Nonuniform System. I. Interfacial Free Energy. The Journal of Chemical Physics. 1958;28(2):258–67.

[34] Rh Stokes RAR. Electrolyte solutions: Mineola NY: Dover Publications; 2002 p. 76 p.

[35] Goulian M, Bruinsma R, Pincus P. Long-Range Forces in Heterogeneous Fluid Membranes. EPL (Europhysics Letters). 1993;22(2):145.

[36] Fischer-Friedrich E, van yen RN, Kruse K. Surface waves of Min-proteins. Phys Biol. 2007;4(1):38–47. 17406084 10.1088/1478-3975/4/1/005

[37] Kang GB, Song HE, Kim MK, Youn HS, Lee JG, An JY, et al Crystal structure of Helicobacter pylori MinE, a cell division topological specificity factor. Mol Microbiol. 2010;76(5):1222–31. Epub 2010/04/20. MMI7160 [pii] 10.1111/j.1365-2958.2010.07160.x 20398219 PMC2883074

[38] King GF, Shih YL, Maciejewski MW, Bains NP, Pan B, Rowland SL, et al Structural basis for the topological specificity function of MinE. Nat Struct Biol. 2000;7(11):1013–7. Epub 2000/11/04. 10.1038/80917 . 11062554

[39] Arjunan SN, Tomita M. A new multicompartmental reaction-diffusion modeling method links transient membrane attachment of E. coli MinE to E-ring formation. Systems and synthetic biology. 2010;4(1):35–53. 10.1007/s11693-009-9047-2 20012222 PMC2816228

[40] Park KT, Wu W, Lovell S, Lutkenhaus J. Mechanism of the asymmetric activation of the MinD ATPase by MinE. Mol Microbiol. 2012;85(2):271–81. 10.1111/j.1365-2958.2012.08110.x 22651575 PMC3376909

[41] Meacci G, Ries J, Fischer-Friedrich E, Kahya N, Schwille P, Kruse K. Mobility of Min-proteins in Escherichia coli measured by fluorescence correlation spectroscopy. Phys Biol. 2006;3(4):255–63. 17200601 10.1088/1478-3975/3/4/003

[42] Berg HC. Random walks in biology. Princeton, N.J: Princeton University Press; 1983 ix, 142 p. p.

[43] Knight JD, Lerner MG, Marcano-Velazquez JG, Pastor RW, Falke JJ. Single molecule diffusion of membrane-bound proteins: window into lipid contacts and bilayer dynamics. Biophys J. 2010;99(9):2879–87. 10.1016/j.bpj.2010.08.046 21044585 PMC2966005

[44] Shih Y-L, Fu X, King GF, Le T, Rothfield L. Division site placement in E.coli: mutations that prevent formation of the MinE ring lead to loss of the normal midcell arrest of growth of polar MinD membrane domains. EMBO J. 2002;21(13):3347–57. 12093736 10.1093/emboj/cdf323 PMC126078

[45] Hu Z, Lutkenhaus J. Topological regulation of cell division in Escherichia coli involves rapid pole to pole oscillation of the division inhibitor MinC under the control of MinD and MinE. Mol Microbiol. 1999;34(1):82–90. . 10540287 10.1046/j.1365-2958.1999.01575.x

[46] Sengupta S, Rutenberg A. Modeling partitioning of Min proteins between daughter cells after septation in Escherichia coli. Phys Biol. 2007;4(3):145–53. 10.1088/1478-3975/4/3/001 . 17928653

[47] Landgraf D, Okumus B, Chien P, Baker TA, Paulsson J. Segregation of molecules at cell division reveals native protein localization. Nat Methods. 2012;9(5):480–2. 10.1038/nmeth.1955 . 22484850 PMC3779060

[48] Sun Q, Margolin W. FtsZ dynamics during the division cycle of live Escherichia coli cells. J Bacteriol. 1998;180(8):2050–6. 9555885 10.1128/jb.180.8.2050-2056.1998 PMC107129

[49] Kelly C. Effect of antimicrobial agents on MinD protein oscillations in Escherichia coli. Available: https://atrium.lib.uoguelph.ca/xmlui/bitstream/handle/10214/3135/thesis-ckelly-17nov2011-final.pdf?sequence=6: The University of Guelph; 2011.

[50] Loose M, Fischer-Friedrich E, Herold C, Kruse K, Schwille P. Min protein patterns emerge from rapid rebinding and membrane interaction of MinE. Nat Struct Mol Biol. 2011;18(5):577–83. 10.1038/nsmb.2037 . 21516096

[51] Suefuji K, Valluzzi R, RayChaudhuri D. Dynamic assembly of MinD into filament bundles modulated by ATP, phospholipids, and MinE. Proc Natl Acad Sci U S A. 2002;99(26):16776–81. 12482939 10.1073/pnas.262671699 PMC139220

[52] Shih Y-L, Le T, Rothfield L. Division site selection in Escherichia coli involves dynamic redistribution of Min proteins within coiled structures that extend between the two cell poles. Proc Natl Acad Sci U S A. 2003;100(13):7865–70. 12766229 10.1073/pnas.1232225100 PMC164679

[53] Ivanov V, Mizuuchi K. Multiple modes of interconverting dynamic pattern formation by bacterial cell division proteins. Proc Natl Acad Sci U S A. 2010;107(18):8071–8. 10.1073/pnas.0911036107 20212106 PMC2889524

[54] Hale CA, Meinhardt H, de Boer PA. Dynamic localization cycle of the cell division regulator MinE in Escherichia coli. EMBO J. 2001;20(7):1563–72. 11285221 10.1093/emboj/20.7.1563 PMC145461

[55] Fu X, Shih YL, Zhang Y, Rothfield LI. The MinE ring required for proper placement of the division site is a mobile structure that changes its cellular location during the Escherichia coli division cycle. Proc Natl Acad Sci U S A. 2001;98(3):980–5. 11158581 10.1073/pnas.031549298 PMC14695

[56] Ghosal D, Trambaiolo D, Amos LA, Lowe J. MinCD cell division proteins form alternating copolymeric cytomotive filaments. Nat Commun. 2014;5:5341 10.1038/ncomms6341 25500731 PMC4338524

[57] Di Ventura B, Knecht B, Andreas H, Godinez WJ, Fritsche M, Rohr K, et al Chromosome segregation by the Escherichia coli Min system. Mol Syst Biol. 2013;9:686 10.1038/msb.2013.44 24022004 PMC3792344

