# Supplementary figures and images for: Molecular Interactions of the Min Protein System Reproduce Spatiotemporal Patterning in Growing and Dividing Escherichia coli Cells
Source: PLoS One. 2015 May 27;10(5):e0128148. doi: 10.1371/journal.pone.0128148 (PMC4446092; doi:10.1371/journal.pone.0128148)

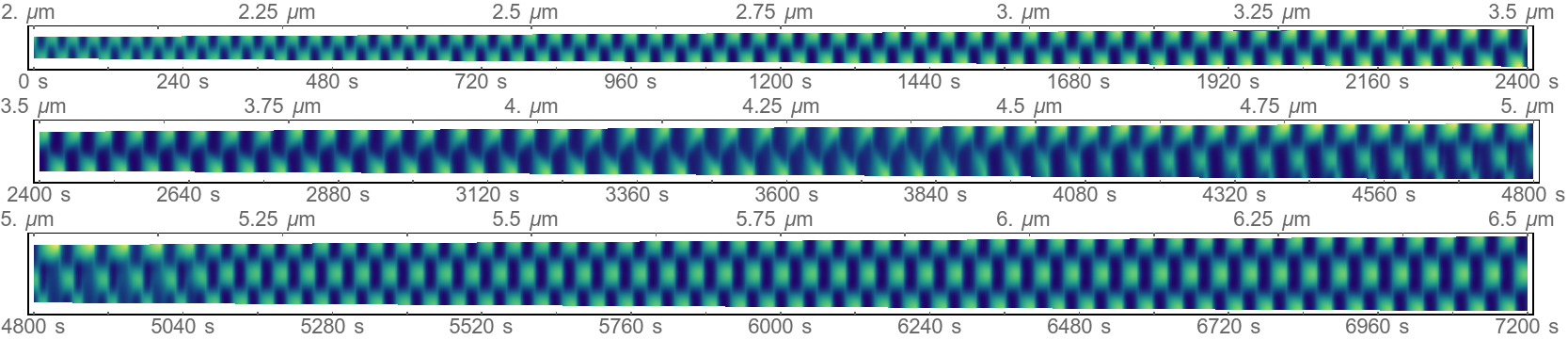

Supplement: S1 Fig — The fully-labelled system was simulated with MinD and MinE each containing a third of their normal concentrations (463 μm -3 (0.76 μM) and 162 μm -3 (0.26 μM), respectively). The resulting kymograph displays no stochastic switching at short cell lengths, with the Min system oscillating from the start of the simulation with a period of approximately 38 s. The system begins to transition away from a pure first order mode at approximately 4000s (with a length of 4.5 μm). This transitional segment contains mid cell antinodes before the second order mode dominates at 5000s (cell length of 5.1 μm). (TIFF) [file pone.0128148.s001.tiff]

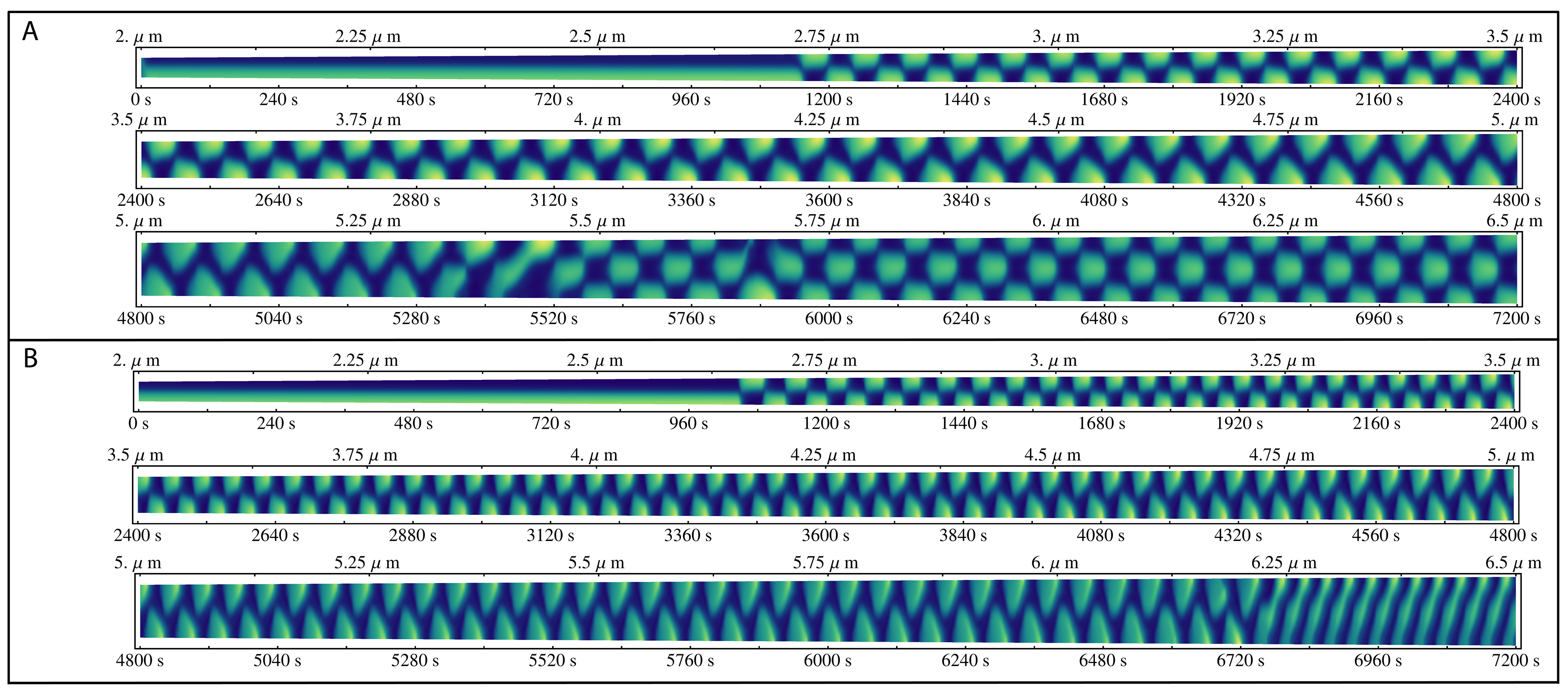

Supplement: S2 Fig — (A) Kymograph of the model used for the results in this paper, the reactions for which are summarised in Fig 1. B) A model with the same basic reactions as (A) except that MinE binding to the membrane is mediated by MinD dimers. That is, MinE in solution (E 2) binds with membrane-bound MinD dimers (d 2) to form a heterotetramer (d 2 e 2) instead of binding directly to the membrane. (TIF) [file pone.0128148.s002.tif]

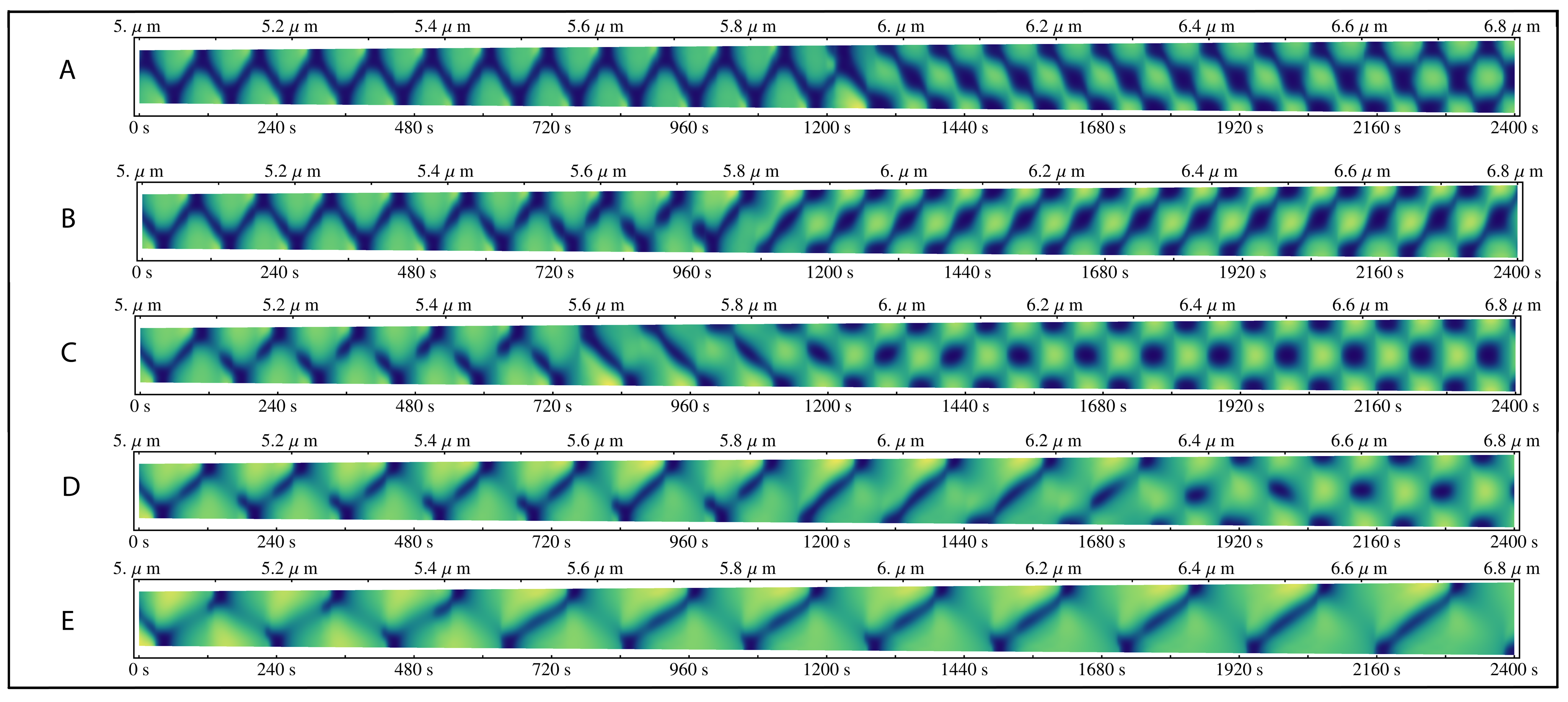

Supplement: S3 Fig — The resulting kymographs from simulations run with (A) 90% (B) 85% (C) 80% (D) 75% (E) 70% of the wild type concentration of MinE. (TIF) [file pone.0128148.s003.tif]

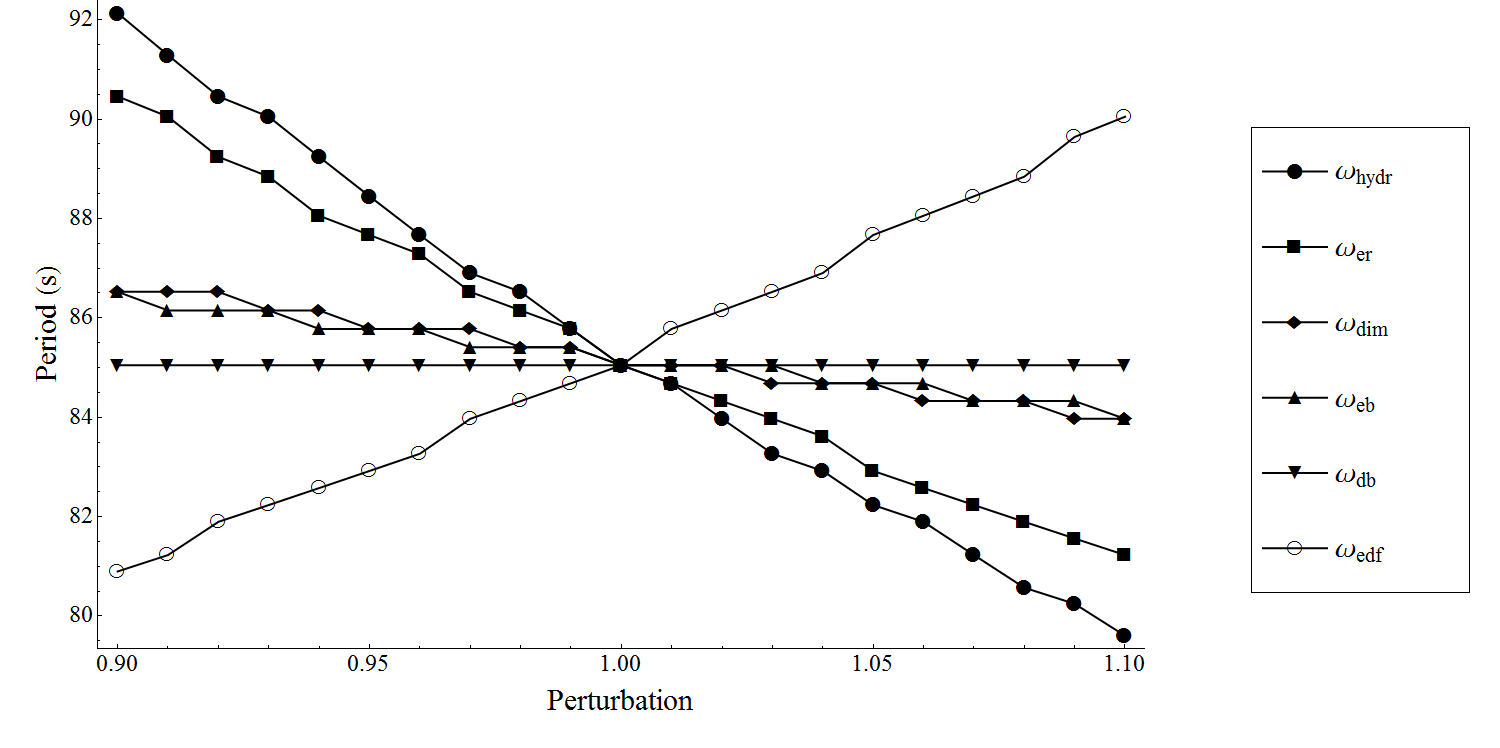

Supplement: S4 Fig — Each parameter is scaled from 0.9 to 1.1 times its original value while the remaining parameters are held constant. The period is then determined by taking the maximum Fourier component of each resulting simulation. (TIF) [file pone.0128148.s004.tif]

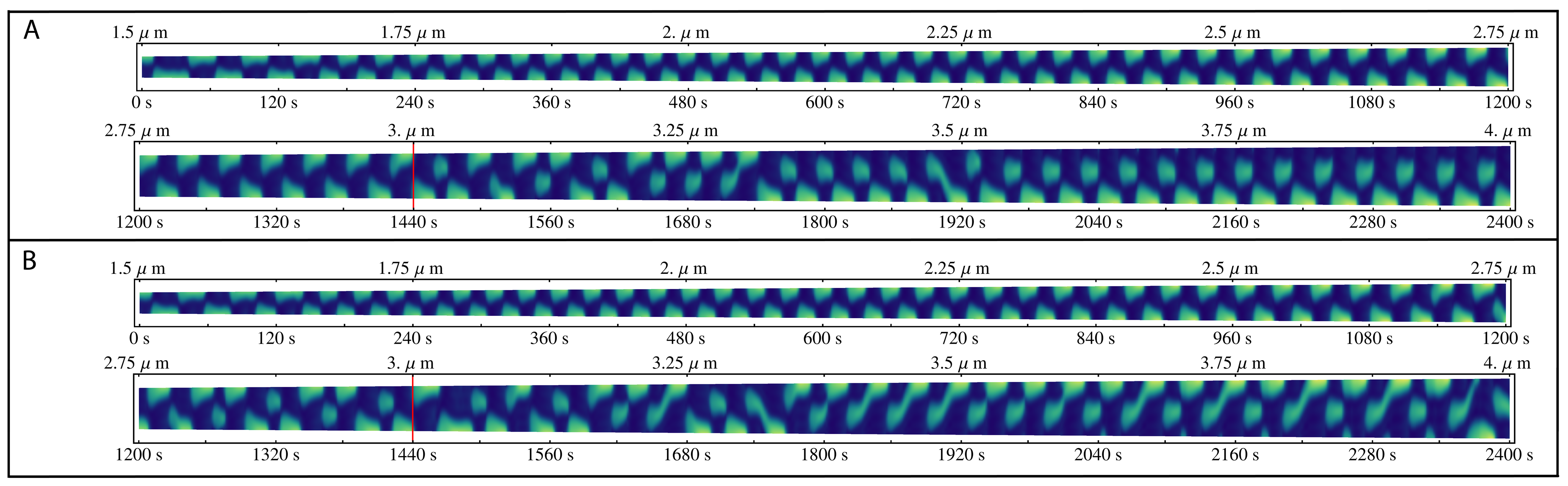

Supplement: S5 Fig — (A) Kymograph of the system with the diffusion of MinD in solution set to 24 μm 2 s -1 (B) Kymograph grown over the same length range with the same parameters except that the MinD in solution is set to the experimental measurement for MinD-GFP of 16 μm 2 s -1 [41]. The experimentally reported length where midcell antinodes occurs (3 μm) is marked with a red line. (TIF) [file pone.0128148.s005.tif]
