# Supplementary material for: Molecular Interactions of the Min Protein System Reproduce Spatiotemporal Patterning in Growing and Dividing Escherichia coli Cells
Source: PLoS One. 2015 May 27;10(5):e0128148. doi: 10.1371/journal.pone.0128148 (PMC4446092; doi:10.1371/journal.pone.0128148)
Supplement: S1 Table — (DOC) [file pone.0128148.s010.doc]

**Table S1. Rate Parameters for Final Model Compared to MinD Mediated MinE Binding Model.**

| Constant | Final Model | MinD Mediated MinE Binding |
| --- | --- | --- |
| *ω_db_* | 4 *μm s^-^*^1^ | 4 *μm s^-^*^1^ |
| *ω_edf_* | 22 *μm*^2^ *s^-^*^1^ | 175 *μm*^2^ *s^-^*^1^ |
| *ω_dim_* | 0.002 *μm*^2^ *s^-^*^1^ | 0.002 *μm*^2^ *s^-^*^1^ |
| *ω_hydr_* | 0.12 *s^-^*^1^ | 0.12 *s^-^*^1^ |
| *ω_eb_* | 0.07 *μm s^-^*^1^ | 0.0475 *μm^3^ s^-^*^1^ |
| *ω_er_* | 30 *s^-^*^1^ | 30 *s^-^*^1^ |
|  | 16 *μm*^2^ *s^-^*^1^ | 16 *μm*^2^ *s^-^*^1^ |
|  | 20 *μm*^2^ *s^-^*^1^ | 20 *μm*^2^ *s^-^*^1^ |
|  | 0.1 *μm*^2^ *s^-^*^1^ | 0.15 *μm*^2^ *s^-^*^1^ |
