# Supplementary material for: Molecular Interactions of the Min Protein System Reproduce Spatiotemporal Patterning in Growing and Dividing Escherichia coli Cells
Source: PLoS One. 2015 May 27;10(5):e0128148. doi: 10.1371/journal.pone.0128148 (PMC4446092; doi:10.1371/journal.pone.0128148)
Supplement: S1 Text — (DOC) [file pone.0128148.s011.doc]

**Supplementary Text S1**

***MinD Mediated MinE Binding Alternative Model***

It has been proposed that the binding of MinE to the membrane could be mediated by membrane-bound MinD. That is, MinE in solution ($E_{2}$) binds with membrane-bound MinD dimers ($d_{2}$) to form a heterotetramer ($d_{2}e_{2}$) instead of binding directly to the membrane.

Adjusting the MinE binding reaction modifies the PDEs that control the model to:

$$\begin{matrix} \partial_{t}D=\delta\left(\left\|\mathbf{x}-\mathbf{M}\right\|\right)\left(\omega_{edf}e_{2}\cdot d+2\omega_{hydr}d_{e}e_{2}-\omega_{db}D\right)+D_{D}\nabla^{2}D \\ \partial_{t}E_{2}=\delta\left(\left\|\mathbf{x}-\mathbf{M}\right\|\right)\left(\omega_{er}e_{2}-\omega_{eb}E_{2}\cdot d_{2}\right)+D_{E}\nabla^{2}E_{2} \\ \partial_{t}d=\omega_{db}D-2\omega_{\dim}d^{2}-\omega_{edf}e_{2}\cdot d+D_{m}\nabla^{2}d \\ \partial_{t}d_{2}=\omega_{\dim}d^{2}-\omega_{edf}e_{2}\cdot d_{2}-\omega_{eb}E_{2}\cdot d_{2}+\frac{1}{2}D_{m}\nabla^{2}d_{2} \\ \partial_{t}e_{2}=-\omega_{er}e_{2}-\omega_{edf}e_{2}\cdot d_{2}+\omega_{hydr}d_{2}e_{2}+\frac{1}{2}D_{m}\nabla^{2}e_{2} \\ \partial_{t}d_{2}e_{2}=\omega_{edf}e_{2}\cdot d_{2}+\omega_{hydr}d_{2}e_{2}+\omega_{eb}E_{2}\cdot d_{2}+\frac{1}{4}D_{m}\nabla^{2}d_{2}e_{2} \\ \end{matrix}$$

The $\omega_{eb}$ parameter was refit. The different reaction dynamics lead to a different proportion of dimeric membrane-bound MinE. To account for this, the value of $\omega_{edf}$was also changed to 175 *μm*^2^ *s^-^*^1^. These changes lead to a shortening in the critical length at which patterning occurred. This was counteracted by increasing the membrane diffusion from 0.1 to 0.15 *μm*^2^ *s^-^*^1^. The full set of parameters used is summarized in Table S1.

Comparing the system where the binding of MinE to the membrane is mediated by MinD dimers (Figure S2B) with the final model in the main paper (Figure S2A, identical to Figure 2 in the main paper), an increase in the second order mode transition length from 5.4 *μm* to 6.25 *μm* was seen*.* The second order mode transition (Figure S2B , >6.25 *μm*) appeared to be more heavily dominated by the first, rather than second order breather mode. This lead to a travelling wave which lacked a definite increase in density at midcell as seen in the final model and experimental data (see main paper, Figure 3Bii and Figure 3Bi respectively). We also note that the period of oscillation is different with the direct MinE binding to MinD having a shorter period. However, this could have been trivially overcome by rescaling simulation time. Otherwise, there was a strong correlation between the models.
