# Supplementary material for: Molecular Interactions of the Min Protein System Reproduce Spatiotemporal Patterning in Growing and Dividing Escherichia coli Cells
Source: PLoS One. 2015 May 27;10(5):e0128148. doi: 10.1371/journal.pone.0128148 (PMC4446092; doi:10.1371/journal.pone.0128148)
Supplement: S2 Text — (DOCX) [file pone.0128148.s012.docx]

**Supplementary Text S2**

***Breakdown of Min Patterning due to Decreasing MinE:MinD Ratio***

To examine the breakdown in patterning of the Min system as the concentration of MinE is reduced, we performed simulations with MinE concentrations ranging between 70% to 90% of the wild type concentration. The results of these simulations are shown in Figure S3.

For each successive kymograph in Figure S3, the MinE concentration decreases by 5%. With this decreasing MinE concentration, the period of oscillation increases, antinodes extend further away from their respective poles, and the length at which the system makes a transition to the second order mode increases.

At MinE concentrations below 75% of wild type (Figure S3D and Figure S3E) the antinodes in the first order mode regime begin to smear together.
