# Supplementary material for: Molecular Interactions of the Min Protein System Reproduce Spatiotemporal Patterning in Growing and Dividing Escherichia coli Cells
Source: PLoS One. 2015 May 27;10(5):e0128148. doi: 10.1371/journal.pone.0128148 (PMC4446092; doi:10.1371/journal.pone.0128148)
Supplement: S3 Text — (DOC) [file pone.0128148.s013.doc]

**Supplementary Text S3**

***Variation of Oscillation Period Due to Parameter Perturbation***

To test the susceptibility of the model's period of oscillation to variations in parameters, each parameter was independently varied from 90% to 110% of its original value in 1% increments. The resulting plot of percentage variation of each parameter versus period is shown in Figure S4.

The variation of three parameters had a significant impact on the period of oscillation ($\omega_{hydr}$*,*$\omega_{er}$and $\omega_{edf}$). The largest absolute gradient is observed for $\omega_{hydr}$which encapsulates ATP hydrolysis and the dissociation of the MinDE heterotetramer releasing MinD into the cytosol. This parameter is negatively correlated with the oscillation period (higher ATPase rates imply shorter periods) as expected if ATP hydrolysis is the rate-limiting step in the cycle.

Thus, the effect of labelling MinD with GFP could be accounted for by altering at least one of the three sensitive parameters. Alteration of $\omega_{er}$ is unlikely as this reaction does not involve MinD. Alteration of $\omega_{edf}$ is possible, but unlikely, as the addition of GFP to MinD would have to enhance its interaction with MinE. Thus, the most likely effect of GFP labelling of MinD is to alter $\omega_{hydr}$.
