# Supplementary material for: Molecular Interactions of the Min Protein System Reproduce Spatiotemporal Patterning in Growing and Dividing Escherichia coli Cells
Source: PLoS One. 2015 May 27;10(5):e0128148. doi: 10.1371/journal.pone.0128148 (PMC4446092; doi:10.1371/journal.pone.0128148)
Supplement: S4 Text — (DOCX) [file pone.0128148.s014.docx]

**Supplementary Text S4**

***Impact of Diffusion Rescaling***

The impact of rescaling MinD diffusion in the minimally-labelled case to compensate for the decrease size of the proteins is shown in Figure S5. In this figure, (A) shows a full simulation of the model with the adjusted MinD diffusion constant (24 *μm*^2^ *s^-^*^1^) whereas (B) shows the results for the experimentally measured MinD-GFP diffusion constant (16 *μm*^2^ *s^-^*^1^). Comparing these two kymographs, we see that in the unadjusted system the onset of midcell pausing occurs slightly earlier (2.8 *μm* versus 3 *μm*) and there is a greater tendency for antinodes to be connected in the midcell pausing regime. Besides these two features, the two systems are very similar.
